# Supplementary material for: Modern Extraction and Purification Techniques for Obtaining High Purity Food-Grade Bioactive Compounds and Value-Added Co-Products from Citrus Wastes
Source: Foods. 2019 Oct 23;8(11):523. doi: 10.3390/foods8110523 (PMC6915388; doi:10.3390/foods8110523)
Supplement: Supplementary file 1 [file foods-08-00523-s001.pdf]

Supplementary Information

Review

Modern extraction and purification techniques for obtaining high purity food grade bioactive compounds and value-added co-products from citrus wastes

Neelima Mahato[1*], Mukty Sinha [2†], Kavita Sharma[3†], Rakoti Koteswararao[2†], Moo Hwan Cho[1]

1 School of Chemical Engineering, Yeungnam University, Gyeongsan, Republic of Korea-38541; neelapchem@gmail.com; mhcho@ynu.ac.kr
2 Department of Medical Devices, National Institute of Pharmaceutical Education and Research, Ahmedabad, Palej, Gandhinagar, India-382 355; muktys@gmail.com, Kingpharmatech@gmail.com
3 Department of Chemistry, Idaho State University, Pocatello 83209, ID, USA; sharkum2@isu.edu
* Correspondence: Neelima Mahato; neelapchem@gmail.com; Tel.: (+82-010-2798-8476)
† Contributed equally as second author

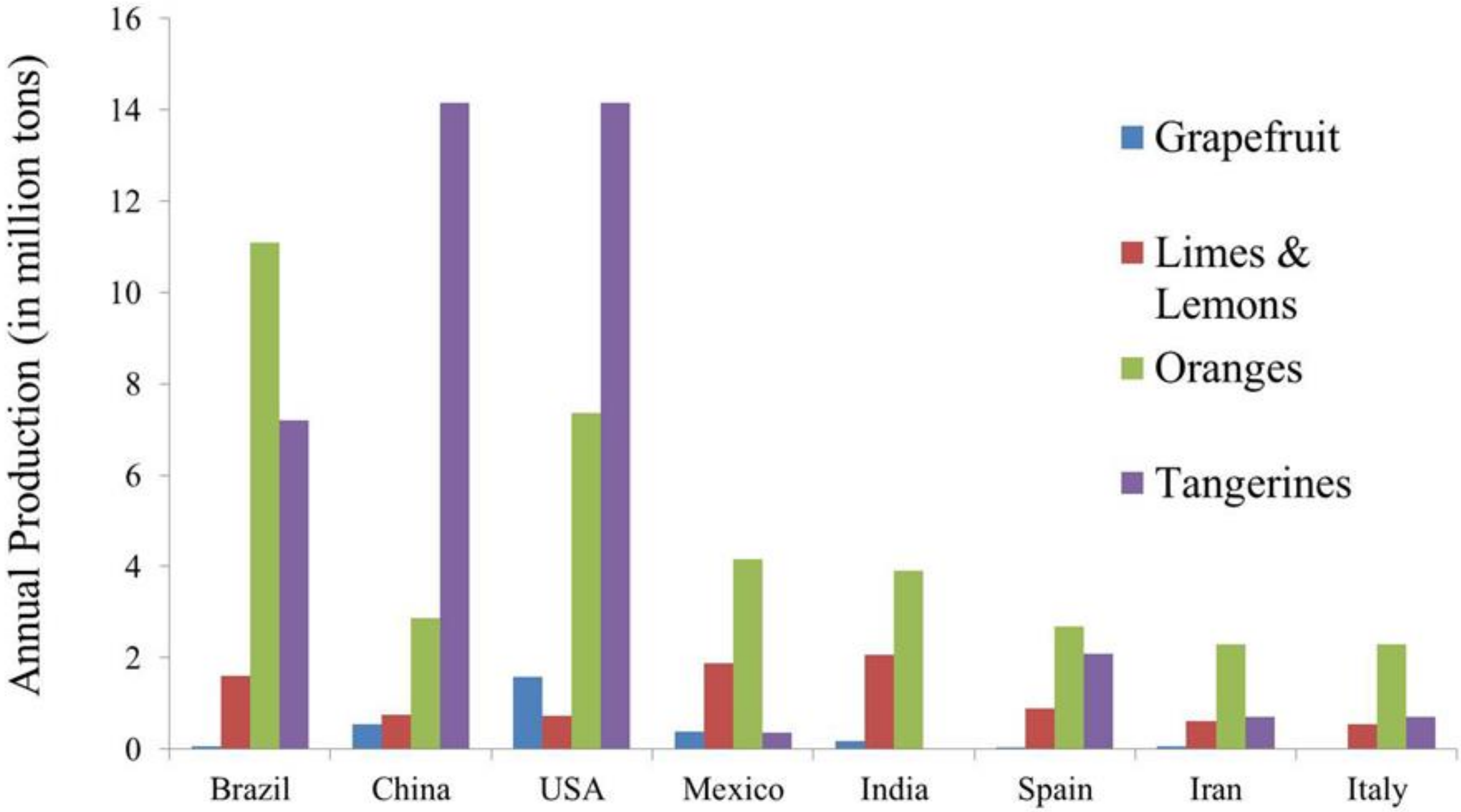

Figure S-1: Major citrus producing countries in the world [1-3].

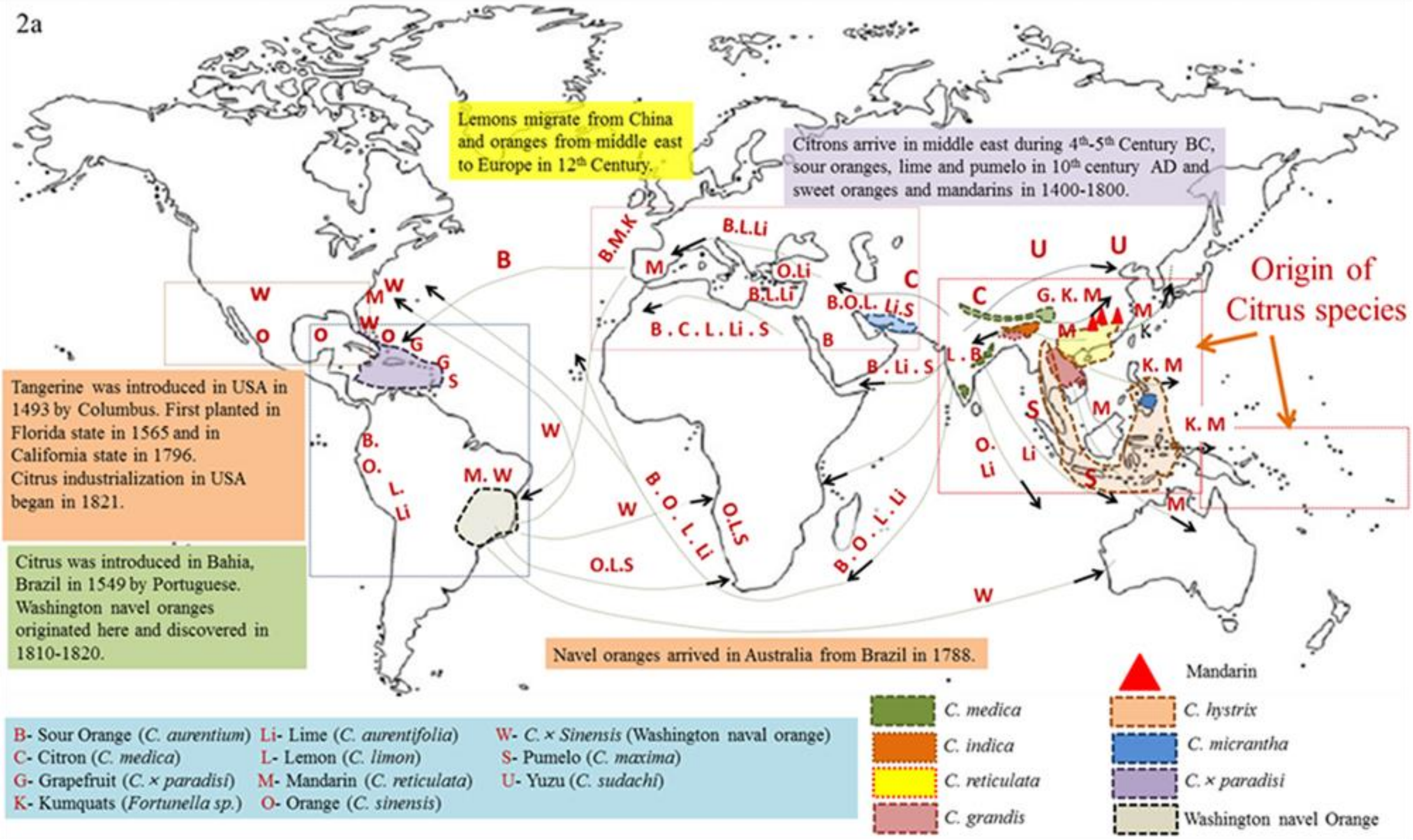
2a
Lemons migrate from China and oranges from middle east to Europe in 12th Century.
Citrons arrive in middle east during 4th-5th Century BC, sour oranges, lime and pumelo in 10th century AD and sweet oranges and mandarins in 1400-1800.
Origin of Citrus species
Tangerine was introduced in USA in 1493 by Columbus. First planted in Florida state in 1565 and in California state in 1796. Citrus industrialization in USA began in 1821.
Citrus was introduced in Bahia, Brazil in 1549 by Portuguese. Washington navel oranges originated here and discovered in 1810-1820.
Navel oranges arrived in Australia from Brazil in 1788.
B.M.K
B.L.Li
O.Li
B.L.Li
B.O.L. Li.S
B . C . L . Li . S
B . Li . S
L . B
G. K. M
K. M
K. M
M. W
B. O. L. Li
O. Li
O.L.S
B . O . L . Li
B . O . L . Li
Mandarin
C. medica
C. indica
C. reticulata
C. grandis
C. hystrix
C. micrantha
C. × paradisi
Washington navel Orange
B- Sour Orange (C. aurentium)
C- Citron (C. medica)
G- Grapefruit (C. × paradisi)
K- Kumquats (Fortunella sp.)
Li- Lime (C. aurentifolia)
L- Lemon (C. limon)
M- Mandarin (C. reticulata)
O- Orange (C. sinensis)
W- C. × Sinensis (Washington naval orange)
S- Pumelo (C. maxima)
U- Yuzu (C. sudachi)

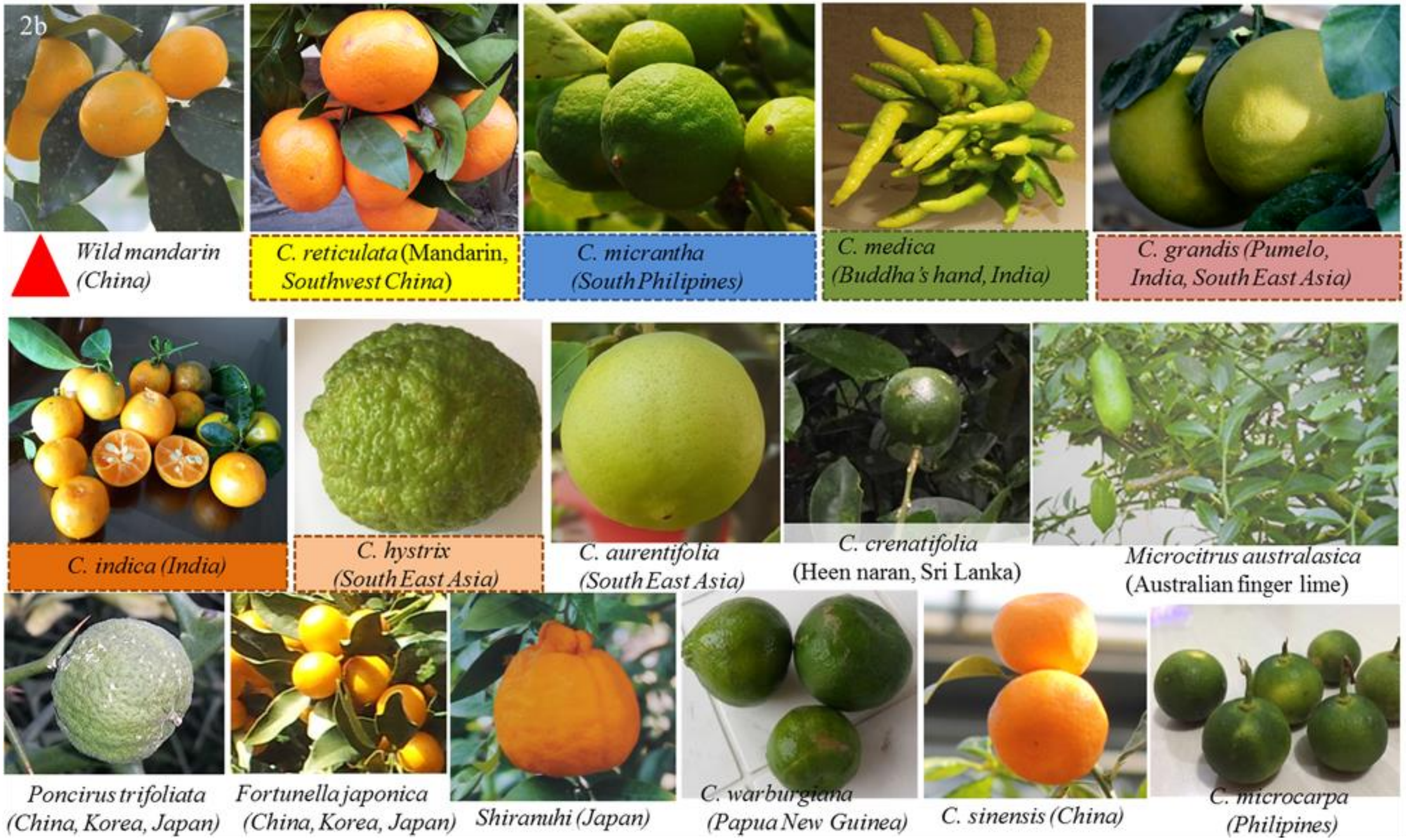
2b
Wild mandarin (China)
C. reticulata (Mandarin, Southwest China)
C. micrantha (South Philipines)
C. medica (Buddha's hand, India)
C. grandis (Pumelo, India, South East Asia)
C. indica (India)
C. hystrix (South East Asia)
C. aurentifolia (South East Asia)
C. crenatifolia (Heen naran, Sri Lanka)
Microcitrus australasica (Australian finger lime)
Poncirus trifoliata (China, Korea, Japan)
Fortunella japonica (China, Korea, Japan)
Shiranuhi (Japan)
C. warburgiana (Papua New Guinea)
C. sinensis (China)
C. microcarpa (Philipines)

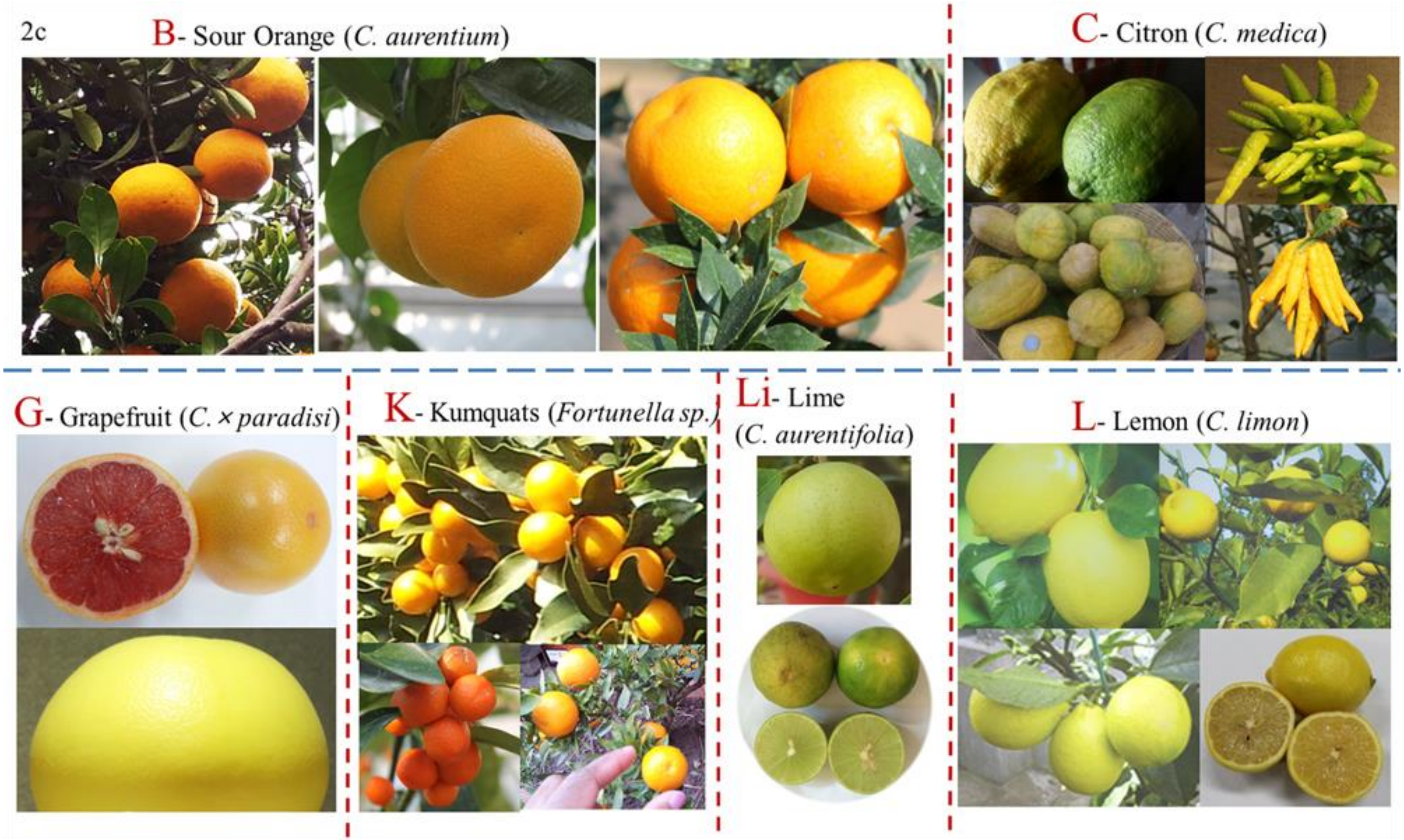
2c
B- Sour Orange (C. aurentium)
C- Citron (C. medica)
G- Grapefruit (C. × paradisi)
K- Kumquats (Fortunella sp.)
Li- Lime (C. aurentifolia)
L- Lemon (C. limon)

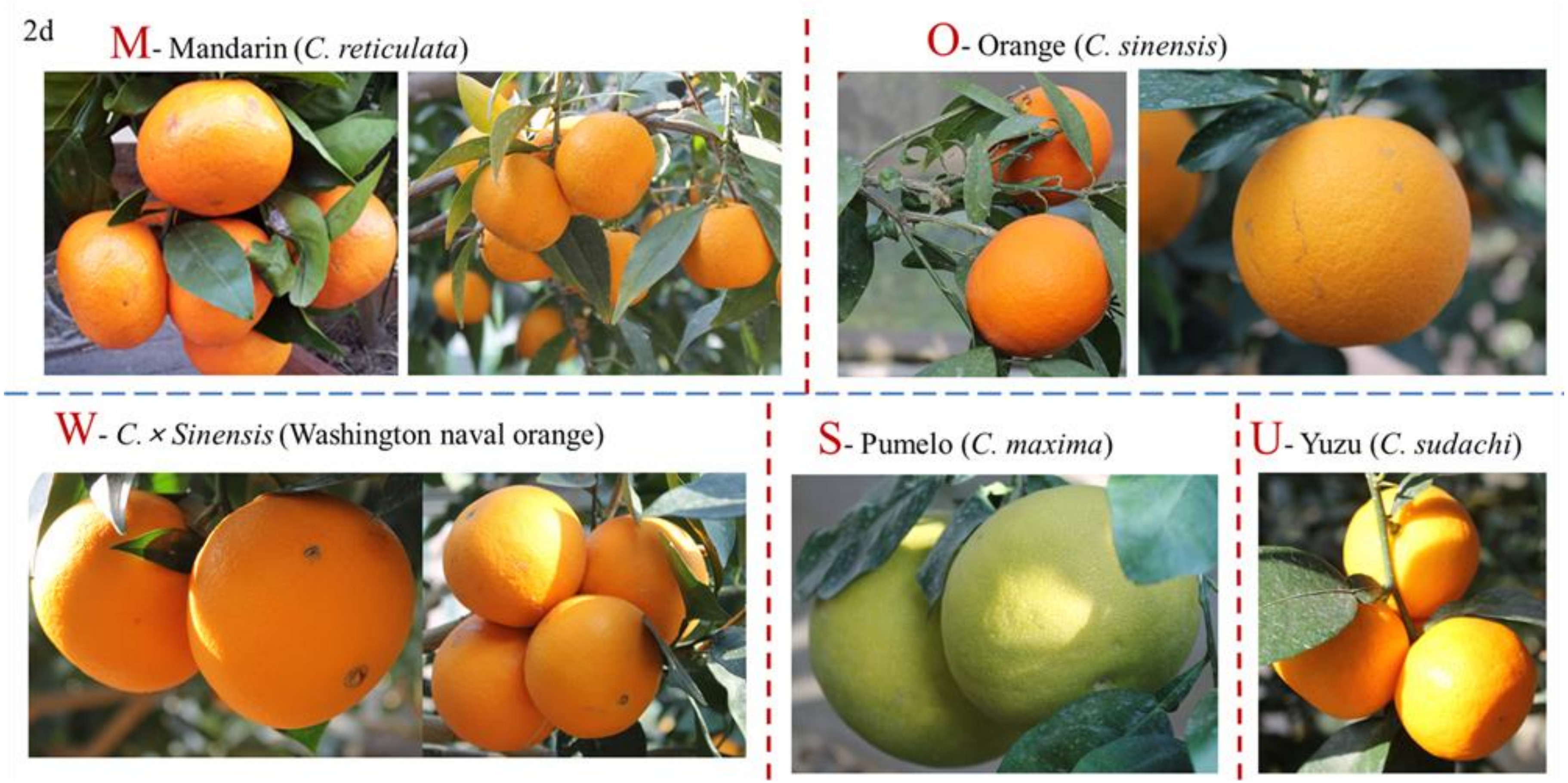

Figure S-2: (a). Origin and spread of citrus fruits across the globe from Himalayan foot hills in India and Southeast regions of China [4,5]; (b): The native citrus fruits, and (c and d): cultivated and hybrid varieties. Photographs are collected from and labelled according to the information available at Sogwipo Citrus Museum, Jeju, South Korea

3a

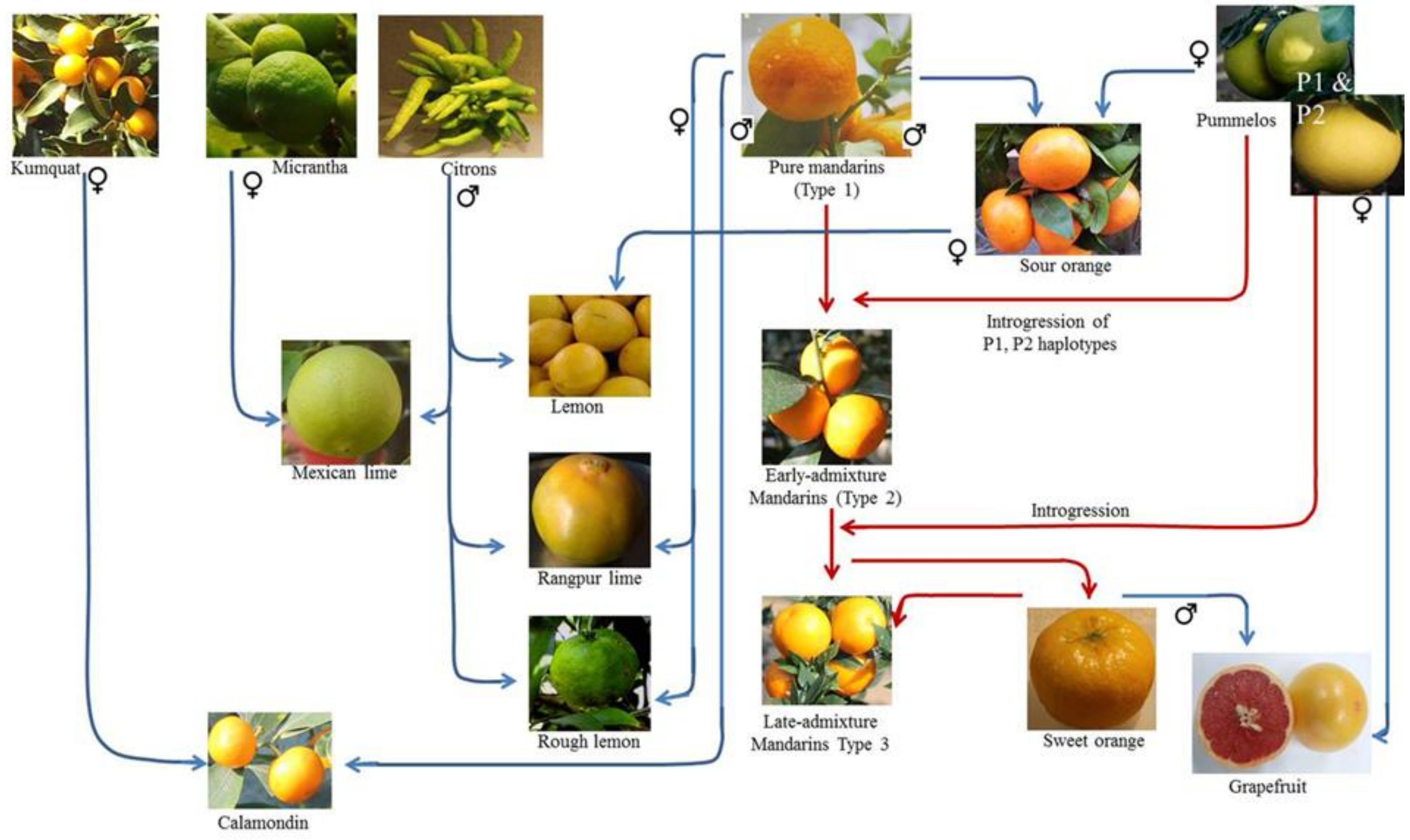
Kumquat
♀
Micrantha
♀
Citrons
♂
♀
♂
Pure mandarins
(Type 1)
♂
♀
Pummelos
P1 &
P2
♀
♀
Sour orange
Introgression of
P1, P2 haplotypes
Lemon
Mexican lime
Early-admixture
Mandarins (Type 2)
Introgression
Rangpur lime
♂
Rough lemon
Late-admixture
Mandarins Type 3
Sweet orange
Grapefruit
Calamondin

3b

Grapefruit<br>*Citrus × paradisi*	A hybrid originating from the cross between two varieties Sweet orange (*C. sinensis*), and Pomelo or Shaddock (*C. maxima*)<br>**Main Varieties**: Oro Blanco, Ruby Red, Pink, Rio Star, Thompson, White Marsh, Flame, Star Ruby, Duncan, Pumelo HB
Oranges<br>*Citrus × sinensis*	A hybrid between Pomelo (*Citrus maxima*) and Mandarin (*Citrus reticulata*)<br>**Main varieties**: The group is subdivided into four distinct classes, *viz.*, Common oranges, Blood or Pigmented oranges, Navel oranges, and Acidless oranges; Mandarin orange (*Citrus reticulata*) is considered to be original species of citrus.<br>Bitter orange (*Citrus aurantium*), Bergamot orange (*Citrus bergamia Risso*), Trifoliate orange (*Poncirus trifoliata*)<br>*Valencia orange*, Hamlin, Blood oranges, Washington or California Navel, Rhode Red, Marrs, Byeonggyul (Jeju, Korea), Cherry orange, Malta, Mosambi, Narinja, Cara cara oranges (red navel)
Lemons<br>*Citrus limon*	A hybrid between Bitter orange (sour orange) and Citron<br>**Main varieties**: Bonnie Brae lemon, Eureka lemon, Femminello St. Teresa', or 'Sorrento, Yen Ben, Flat lemon, Meyer lemon, Ponderosa lemon, Rough lemon, Sweet lemons, Volkamer lemon, Lumia (pear lemon), Limetta, Palestinian sweet lime, Jaffa lemon
Lime<br>*Citrus aurantifolia*	Hybrid from a variety of citrus species, e.g., Citron (*Citrus medica*), Mandarin orange (*Citrus reticulata*), Pomelo (*Citrus maxima*) with many other lime varieties including Micrantha (*Citrus micrantha*)<br>**Main varieties**: Kaffir lime, Persian lime, Kaffir lime, Desert lime, Rangpur lime, Limequat , Sweet lime, Blood lime, Australian lime, Australian finger lime, Australian desert lime, Sweet lime
Pomelo<br>*Citrus maxima*<br>or *Citrus grandis*	It is a natural and non-hybrid citrus fruit<br>**Main varieties** (names): Dangyuja, Banpeiyu, Jabong, Jambola, Shaddock
Yuzu<br>*Citrus junos*	A possible cross between mandarin and papeda or a cross between lime and citron<br>**Main Varieties** (names): Yuzu (Japan) Yuza (Korea), Xiāngchéng (China), Shishi yuzu or Lion yuzu, Hana yuzu, Yuko (sweet yuzu)

Figure S-3: (a) Cross between the native varieties and evolution of hybrid variants in citrus, and (b) list of main citrus varieties cultivated globally [4,6]

Table S-1: General description and principle of different methods and techniques used in the extraction of valuable compounds from citrus

Method	Principle	Working condition	Ref.
**Conventional solvent extraction**			
**Limitations:**<br>• Longer extraction time, requirement of costly and high purity solvent,<br>• Evaporation of the huge amount of solvent, low extraction selectivity<br>• Thermal decomposition of thermolabile compounds			
Soxhlet Extraction	➢ For extracting valuable bioactive compounds from various natural sources.<br>➢ A small amount of dry sample is placed in a thimble, thimble is then placed in distillation flask which contains the solvent of particular interest; After reaching to an overflow level, the solution of the thimble-holder is aspirated by a siphon; Siphon unloads the solution back into the distillation flask.<br>➢ The solution carries extracted solutes into the bulk liquid.<br>➢ Solute remained in the distillation flask and solvent passes back to the solid bed of plant.<br>➢ The process runs repeatedly until the extraction is complete	Water as solvent, temperature 100° C; duration 6 – 10 h	[7], [8]
Maceration	➢ Grinding of plant materials into small particle to increase the surface area for proper mixing with solvent.<br>➢ In the process, appropriate solvent (menstruum) is added in a closed vessel.	At room temperature; solvent – methanol, ethanol, water; duration 10-24 h	[7]

	➢ The liquid is strained off but the marc which is the solid residue of this extraction process is pressed to recover large amount of occluded solutions.<br>➢ The obtained strained and the press out liquid are mixed and separated from impurities by filtration.<br>➢ Occasional shaking in maceration facilitates extraction by increase diffusion, and removes concentrated solution from the sample surface to menstruum for more extraction yield.		
Hydrodistillation (HD)	➢ For extraction of bioactive compounds and essential oils from plants.<br>➢ Performed before dehydration of plant materials.<br>➢ 3 types of HD- water distillation, water and steam distillation and direct steam distillation<br>➢ The plant materials are packed in a still compartment; water is added in sufficient amount and then brought to boil. OR, direct steam is injected into the plant sample.<br>➢ Hot water and steam act as the main influential factors to free bioactive compounds of plant tissue.<br>➢ Indirect cooling by water condenses the vapor mixture of water and oil. Condensed mixture flows from condenser to a separator, where oil and bioactive compounds separate automatically from the water<br>➢ HD involves 3 physicochemical processes; Hydro-diffusion, hydrolysis and decomposition by heat.<br>➢ Limited for thermolabile compound extraction.	Only water used as solvent, 90 – 100° C; Organic solvents are not involved	[9], [10]

Non-conventional extraction techniques

Ultrasound-assisted extraction (UAE)	➢ Waves pass through a medium by creating compression and expansion; produces cavitation, which means production, growth and collapse of bubbles.<br>➢ A large amount of energy can produce from the conversion of kinetic energy of motion into heating the contents of the bubble at high temperature<br>➢ The extraction mechanism involves two main types of physical phenomena, (a) the diffusion across the cell wall and (b) rinsing the contents of cell after breaking the walls	Sound wave in range of 20 kHz to 100 MHz; Working temperature 5000 K; pressure 1000 atm; heating and cooling rate above1010 K/s.	[11], [12], [13]
Pulsed-electric field extraction (PEF)	➢ Suspension of a living cell in electric field, an electric potential passes through the membrane of that cell; based on the dipole nature of membrane molecules, electric potential separates molecules according to their charge in the cell membrane.<br>➢ After exceeding a critical value of approximately1 V of transmembrane potential, repulsion occurs between the charge carrying molecules that form pores in weak areas of the membrane and causes drastic increase of permeability	Electric field -500 and 1000 V/cm; for 10^{-4}–10^{-2} s very less increase in temperature	[14], [15], [16]
Enzyme-assisted extraction (EAE)	➢ For compounds retained in the polysaccharide-lignin network by hydrogen or hydrophobic bonding<br>➢ The addition of specific enzymes like cellulase, a-amylase, and pectinase during extraction enhances recovery by breaking the cell wall and hydrolyzing the structural polysaccharides and lipid bodies<br>➢ two approaches for enzyme-assisted extraction: (1) enzyme-assisted aqueous extraction (EAAE) and (2) enzyme-assisted cold pressing (EACP)<br>➢ EAAE methods for the extraction of oils from various seeds	Enzymes used for extraction: cellulase, a-amylase, and pectinase, at room temperature	[17,18] [19-25]

	➢ In EACP technique, enzymes is used to hydrolyze the seed cell wall,<br>➢ enzyme composition and concentration, moisture content of plant materials, particle size of plant materials, solid to water ratio, and hydrolysis time are key factors for extraction<br>➢ eco-friendly technology for extraction of bioactive compounds and oil as water used as solvent		
Microwave assisted extraction	➢ The principle of heating is based upon its direct impacts on polar materials; Electromagnetic energy is converted to heat following ionic conduction and dipole rotation mechanisms; During ionic conduction mechanism heat is generated because of the resistance of medium to flow ion.<br>➢ Due to ionic conduction and movement heat is generated<br>➢ The extraction involve three sequential steps; first, separation of solutes from active sites of sample matrix under increased temperature and pressure; second, diffusion of solvent across sample matrix; third, release of solutes from sample matrix to solvent.<br>➢ Advantages: quicker heating for the extraction of bioactive substances from plant materials	Microwaves frequency range 300 MHz to 300 GHz.	[26,27]
Pressurized liquid extraction /pressurized fluid extraction / accelerated fluid extraction (ASE)/ enhanced solvent	➢ Application of high pressure to remain solvent liquid beyond their normal boiling point. High pressure facilitates the extraction process.<br>➢ The higher extraction temperature can promote higher analyte solubility by increasing both solubility and mass transfer rate and, also decrease the viscosity and surface tension of solvents, thus improving extraction rate<br>➢ decrease time consumption and solvent use; preferred for extraction of polar compounds	Ethanol and water (70:30) at 50–150 °C; water at 50–130 °C	[28-31]

extraction (ESE)/ and high pressure solvent extraction (HSPE)			
Supercritical Fluid Extraction (SFE)	➢ Supercritical fluid possesses gas-like properties of diffusion, viscosity, and surface tension, and liquid-like density and solvation power, suitable for extracting compounds in a short time with higher yields<br>➢ The system consists of a tank of mobile phase, CO_2, a pump to pressurize the gas, co-solvent vessel and pump, an oven containing extraction vessel, a controller to maintain the high pressure inside the system and a trapping vessel.	CO_2 (31 °C); pressure 100 and 450 bar	[32-36]
Sub Critical water (SCW) extraction	➢ SCW have high density, high reactivity, and good solubility for a series of organic compounds and high catalytic activity.<br>➢ Citrus fruit with distilled water placed in a vessel which can withstand the pressure, after tightly closing, the vessel was placed in an extractor, the extraction performed in SCW at given temperature range and pressure.<br>➢ After achieving desired conditions, the vessel immediately and taken out from the oven and cooled to room temperature.<br>➢ Then the extracts centrifuged and the supernatants stored at 4°C.	Hot water; temperature range 100 and 374°C under high pressure to maintain its liquid state (critical point of water, 22.4MPa and 374°C)	[37,38]
Microwave Steam Distillation or microwave 'dry' distillation	➢ Used to obtain essential oils from aromatic herbs<br>➢ Involves placing fresh vegetable material in a microwave reactor. The internal heating of the in situ water within the plant material distends it and makes the glands and oleiferous receptacles burst.	Microwave irradiation power: 135W- 445W; time 5-10 min	[39,40]

(MSD)	➢ This process thus frees essential oil, which is entrained by the in situ water of the plant material by azeotropic distillation.<br>➢ The vapor then passes through a condenser outside the microwave cavity, where it condensed.<br>➢ The distillate is collected continuously in a receiving flask.		
Cold Pressing	➢ The epidermis and oil glands lacerated with a needle, creating areas of compression in the peel surrounded by areas of lower pressure, across which the oil flows to the exterior.<br>➢ The oil was carried down to a decantation vessel in a stream of water, the emulsion collected and separated by centrifugation.<br>➢ The essential oil collected, dried over anhydrous sodium sulphate and stored at 4 °C until used.	Low pressure, room temperature, water used as solvent	[39,41]
Simultaneous saccharification and fermentation (SSF)	➢ The technique used for the production of ethanol from Citrus waste;<br>➢ It combines enzymatic hydrolysis with fermentation in the same vessel at the same time.<br>➢ Enzymes hydrolyze polysaccharides into sugars which immediately consumed by yeast to produce ethanol.<br>➢ Hydrolysis rates increases by reducing product inhibition of enzymes and reduces container usage by combining the saccharification and fermentation into one tank.<br>➢ Widely used in the dry grind corn ethanol industry	*Saccharomyces cerevisiae* yeast and *Escherichia coli* Bacteria; 10–12 rpm at 37 °C.	[42]
Supercritical CO_2 (SC-CO_2) extraction	➢ Ultrasonic techniques can enhance SC-CO_2 extraction<br>➢ Both extraction method applied together<br>➢ Yield is more compare to individual process	Ultrasonic power outputs 0 to 400W; maximal	[43]

enhanced by ultrasound		resistant pressure 35MPa; temperature 55 ◦C	
Microwave hydro-diffusion and gravity (MHG)	➢ Combination of microwave heating and gravity working at atmospheric pressure. The plant material is directly placed in a microwave reactor without any added solvent or water; heating of thein situ water within the plant material distends the plant cells and rupture of the glands and cell receptacles; heating frees molecules of interest together with in-situ water, i.e., hydro-diffusion, allows the extract to diffuse outside the plant material and<br>➢ Extract drop by earth gravity out of the microwave reactor through the perforated Pyrex disc.	No solvent used; microwave power 500W for15 min	[44]

4a

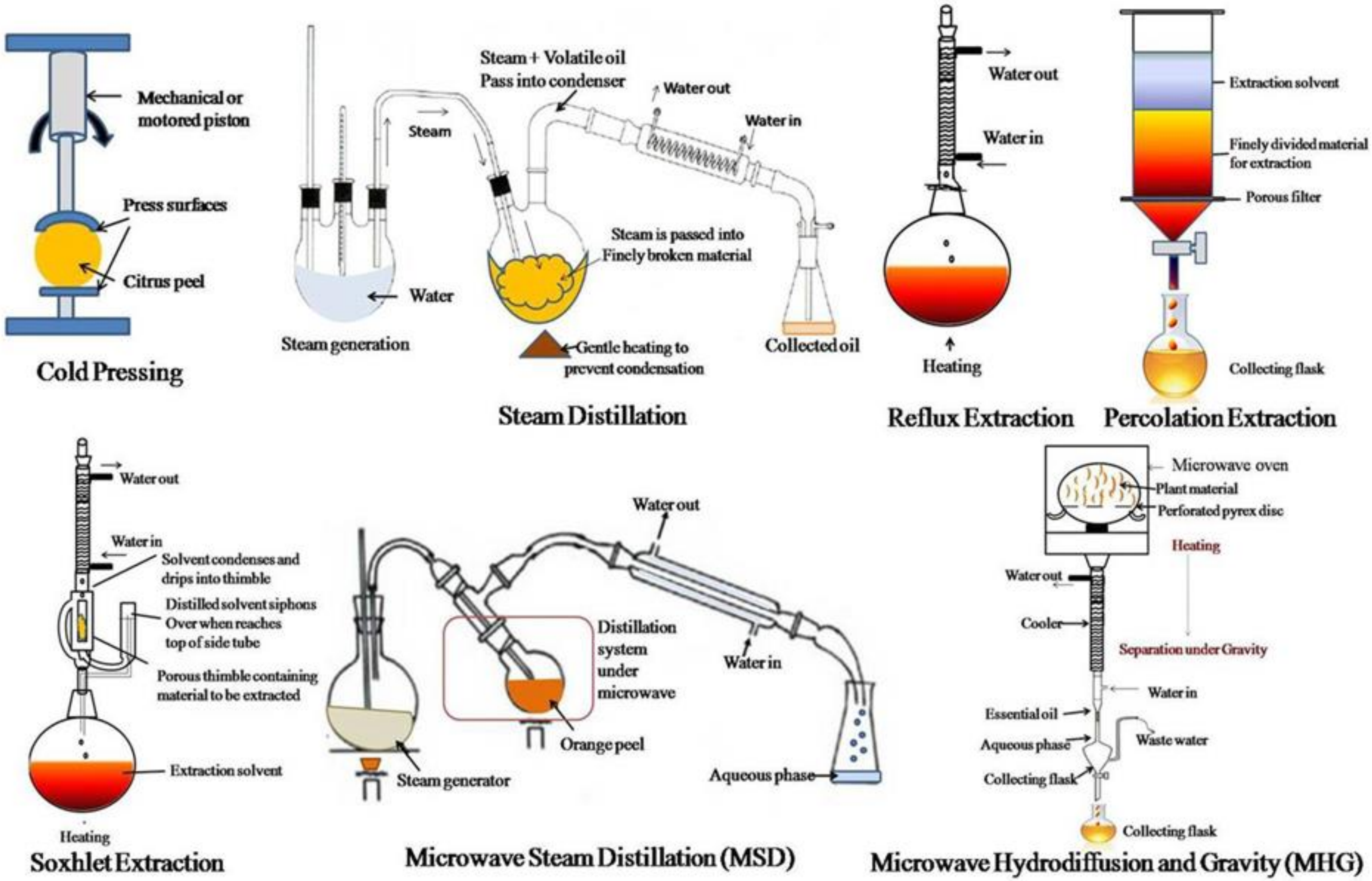
Mechanical or motored piston
Press surfaces
Citrus peel
Cold Pressing
Steam + Volatile oil Pass into condenser
Water out
Water in
Steam
Steam is passed into Finely broken material
Water
Steam generation
Gentle heating to prevent condensation
Collected oil
Steam Distillation
Water out
Water in
Heating
Reflux Extraction
Extraction solvent
Finely divided material for extraction
Porous filter
Collecting flask
Percolation Extraction
Water out
Water in
Solvent condenses and drips into thimble
Distilled solvent siphons Over when reaches top of side tube
Porous thimble containing material to be extracted
Extraction solvent
Heating
Soxhlet Extraction
Water out
Distillation system under microwave
Water in
Orange peel
Steam generator
Aqueous phase
Microwave Steam Distillation (MSD)
Microwave oven
Plant material
Perforated pyrex disc
Heating
Water out
Cooler
Separation under Gravity
Water in
Essential oil
Aqueous phase
Waste water
Collecting flask
Collecting flask
Microwave Hydrodiffusion and Gravity (MHG)

4b

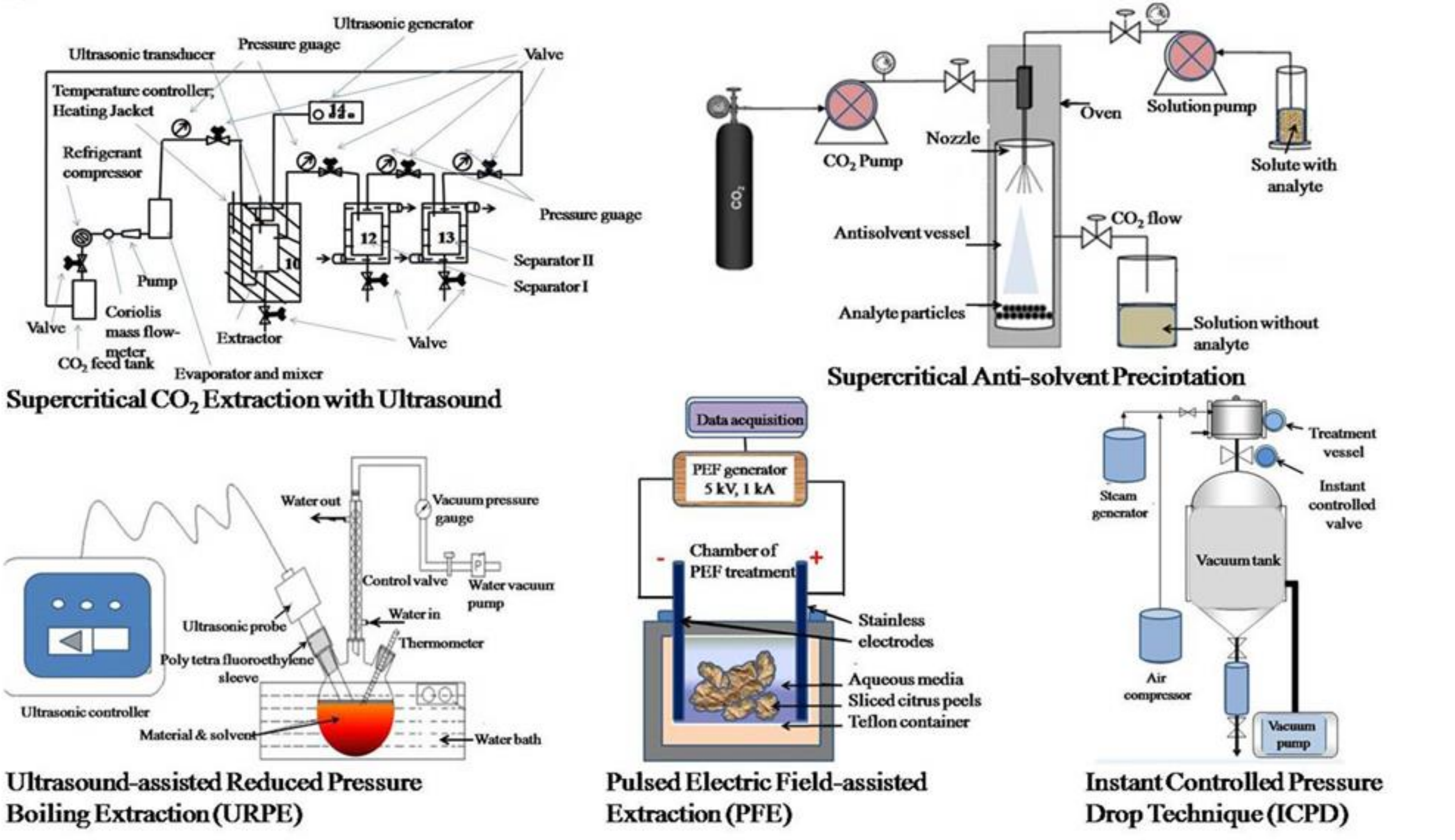

Ultrasonic generator
Ultrasonic transducer
Pressure guage
Valve
Temperature controller, Heating Jacket
Refrigerant compressor
Pressure guage
Separator II
Separator I
Pump
Valve
Coriolis mass flow-meter
Extractor
Valve
CO2 feed tank
Evaporator and mixer
Supercritical CO2 Extraction with Ultrasound
CO2
CO2 Pump
Oven
Solution pump
Nozzle
Solute with analyte
Antisolvent vessel
CO2 flow
Analyte particles
Solution without analyte
Supercritical Anti-solvent Preciptation
Water out
Vacuum pressure gauge
Control valve
Water vacuum pump
Water in
Ultrasonic probe
Thermometer
Poly tetra fluoroethylene sleeve
Ultrasonic controller
Material & solvent
Water bath
Ultrasound-assisted Reduced Pressure Boiling Extraction (URPE)
Data acquisition
PEF generator 5 kV, 1 kA
Chamber of PEF treatment
Stainless electrodes
Aqueous media
Sliced citrus peels
Teflon container
Pulsed Electric Field-assisted Extraction (PFE)
Treatment vessel
Steam generator
Instant controlled valve
Vacuum tank
Air compressor
Vacuum pump
Instant Controlled Pressure Drop Technique (ICPD)

4c

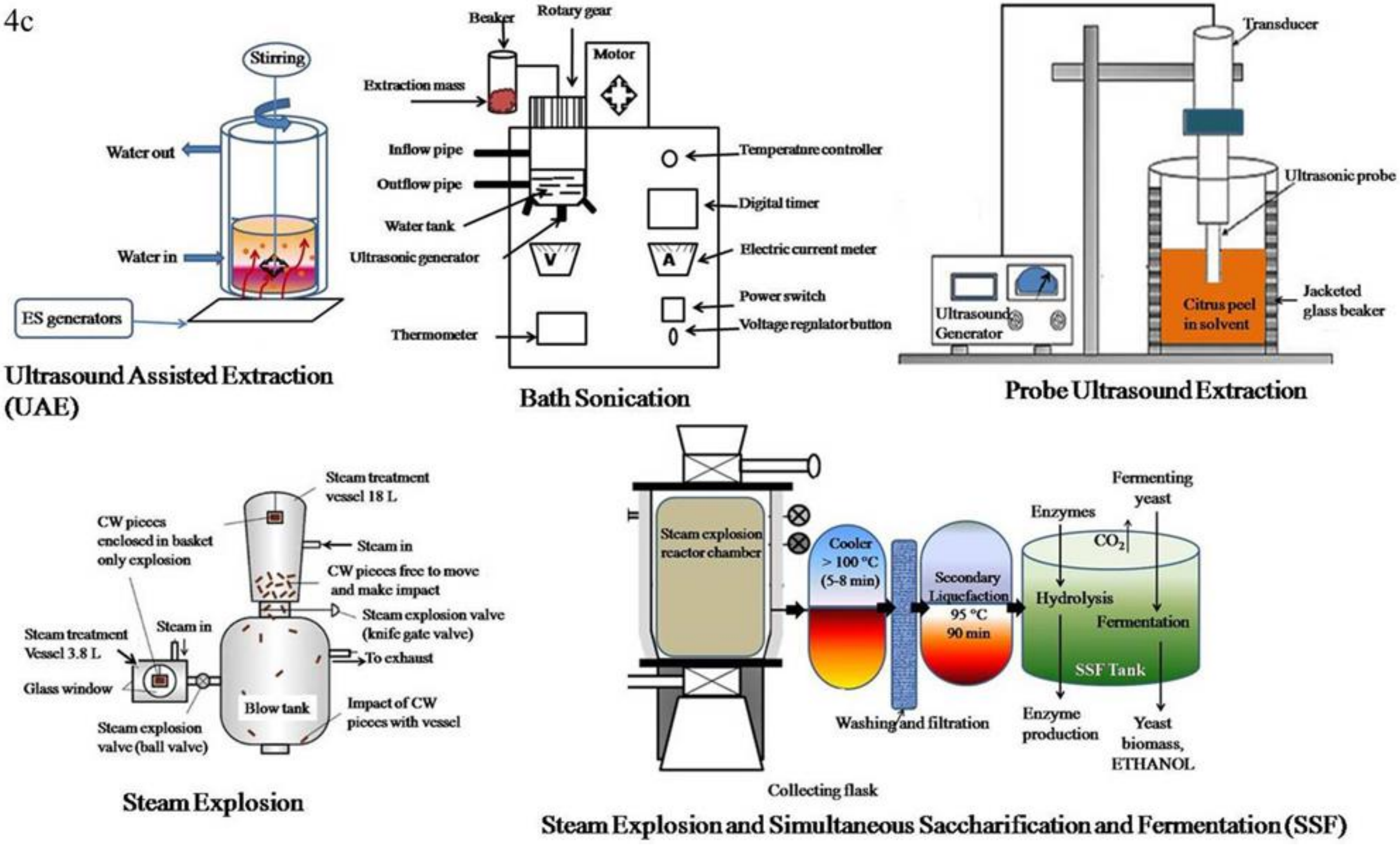

Figure S-4. Schematic representation of different extraction techniques

Table S-2: Estimation and analysis of the products obtained from extraction

Type of activity	Method of estimation	Expressed in Units	Ref
Total Phenolics by Folin-Ciocalteu spectrophotometric method	Diluted extract of orange, distilled water and Folin–Ciocalteau reagent (2 N) added; after 5 min of incubation at room temperature, solution of Na_2CO_3 (2% *v*/*v*) and distilled water added to the mixture and incubated for 90 min; after incubation, absorbance measured at 750 nm.	mg of gallic acid equivalents (GAE) per 100 g of weight of orange peel.	[37,45, 46]
**Antioxidant activity**			
Ferric Reducing Ability Assay (FRAP)	FRAP reagent prepared as a mixture of 0.1 M acetate buffer (pH 3.6), 10 mM of 2,4,6-tris(2-pyridyl)-s-triazine, and 20 mM ferric chloride (10:1:1, *v*/*v*/*v*). For the assay, 1.9 mL of reagent added to 0.1 mL of extract. Absorbance at 593 nm, measured;6-hydroxy-2,5,7,8-tetramethylchroman-2-carboxylic acid (Trolox) solution used to perform the calibration curves.	Trolox equivalent antioxidant capacity (TEAC) milligrams per gram of DW	[46,47]
2,2-azinobis-(3-ethylbenzothiazoline-6-sulfonate) (ABTS)Free Radical Scavenging Assay	A decolorization assay; To oxidize the colorless ABTS to the blue-green ABTS radical cation, ABTS (7 mM) mixed with potassium persulfate and kept for 12-16 h at room temperature in the dark; for the analysis, the ABTS solution diluted with ethanol; the diluted ABTS solution added to the extract (diluted 5 times by 80 % methanol), the mixture stirred for 30 s and allowed to stand for 15 min at room temperature, and then the absorbance reading determined at 734 nm.	Trolox equivalent antioxidant capacity (TEAC) milligrams per gram of DW	[47]
Reduction of Molybdenum	Total antioxidant capacity measurement based on the ability of potent antioxidant to reduce molybdenum ions. The results are presented as IC_{50}	IC_{50} values	[48]

	values that indicate the concentration of extracts that reduces the 50% of molybdenum. Catechin used as standard probe.		
2,2-dephenyl-1-picrylhydrazyl (DPPH) Radical Scavenging Activity	After mixing the citrus extract with DPPH radical in ethanol for 10 min, the absorbance of the sample measured at 517 nm. The Radical Scavenging Activity expressed as percentage according to the following formula: % RSA= (1 – sample OD/control OD)×100.	Percentage utilization of DPPH radical	[37]
Reducing Power	The Citrus extract, phosphate buffer (pH 6.6), and potassium ferricyanide solution mixed and incubated at 50 °C for 20 min. A trichloroacetic acid solution added to the mixture and centrifuged. The resulting supernatant (1.0 ml) mixed with distilled water (1.0 ml) and a ferric chloride solution (0.1 ml), and then the absorbance measured at 700 nm.	Reduced concentration of Fe^{3+} ions	[37]
Hydroxyl radical assay	Hydroxyl radicals obtained by the Fenton reaction and detected by spin trapping in a system consisting of H_2O_2 (2 mM), $FeCl_2$ (0.3 mM), DMF and 5,5-dimethyl-1-pyroline-Noxide, DMPO (112 mM) as control sample. The influence of extract on the amounts of hydroxyl radicals trapped by DMPO is studied by adding the DMF solution of the extract to the reaction system in the concentration range of 0.05 – 2.0 mg/ml. ESR spectra recorded 2.5 min after mixing on an ESR spectrometer. The scavenging Activity of • OH(SA.OH) value of the extract defined as: SA• OH (%)= 100 × (H_0 – H_x) / H0, Where, H_0 and H_x are the height of the second peak in the ESR spectrum of DMPO/•OH spin adduct of the samples without and with extract, respectively.	Percentage	[49]

Thiobarbituric Acid-Reactive Substances (TBARS)	Lipid oxidation of sample assessed by 2-thiobarbituric acid Method: An aliquot of sample homogenized with Tricoloro acetic acid (5 %) and butylated hydroxyanisole (BHA) (0.8 %) on an ultrasonic bath for 5 min and then centrifuged for 5 min at 3000 rpm; the supernatant added to TBA (0.8 %) and heated in water bath (70 °C) for 30 min for pink color development. The tube cooled and then the absorbance measured at 532 nm. TBARS calculated from a standard curve of malondialdehyde freshly prepared by acidification of ,1,3,3-tetraetoxypropane in the range from 0.006 to 0.299 μg/ml.	mg of malondialdehyde per kg of sample	[49] [50]
Peroxide value (PV)	The lipid samples dissolved in glacial acetic acid: chloroform (3/2 *v*/*v*), and KI solution (14-g KI/10 mL distilled water) added; the mixture titrated against 0.01 N sodium thiosulphate with the presence of starch as an indicator. Peroxide value calculated as:<br>PV(meq. peroxide O_2/kg lipid)= (V – B × Nf/W) × 1000;<br>Where, V = amount of thiosulphate, B = spent thiosulphate for the blank, W = weight of the sample (g), Nf = the normality for sodium thiosulphate.	meq. peroxide O_2/kg lipid	[51]
Superoxide radical scavenging power	Peel extract at different concentrations (25-400 mg/mL) added to 1 mL Na_2CO_3 (5 %), 0.3 mL EDTA (0.5%), and 0.4 mL nitrobluetetrazolium (NBT). The absorbance of the mixture measured immediately at 560 nm. The reaction initiated by the addition of 0.4 mL hydroxlylamine hydrochloride and incubated at 25 °C for 5 minutes; NBT reduction determined with a spectrophotometer at 560 nm. A parallel control (without extract) and standard ascorbic acid analyzed in a similar manner. The percent scavenging activity calculated as follows:<br>$\text{Percentage inhibition of superoxide radical} = \left[1 - \frac{A1}{A0}\right] \times 100$	Percentage inhibition of superoxide radical	[52]

	Where, A1 is the absorbance of extract sample and A0is the absorbance of control.		
Lipolytic Effects	Sample mixed with 900 μL of chloroform and 50 μL of olive oil in a tightly screwed cap vessel. The control sample is prepared using chloroform instead of peel oils or authentic compounds. 4-(4-Hydroxyphenyl)-2-butanone (raspberry ketone) is employed as a standard compound to examine the lipolytic effects; 20 mM 4-(4-hydroxyphenyl)-2-butanone in chloroform added to the reaction mixture (final concentration of 1 mM); The mixture is shaken and left to stand for 60 min at 37 °C in an incubator. After 60 min, the sample subjected to GC analysis. The lipolytic effect is investigated by evaluating the increase of peak area at the gas chromatogram.	Percent of oleic acid	[53]
Antifouling agent by mussels inhibitory effect test	The test is done on mussels (*Mytilus edulis*), a leading shellfish. The test is performed to evaluate the adhesion inhibiting effect on the shellfish, which cannot adhere to the surface in presence of the extract. The percentage shrinkage of roots of shell mussels is recorded.	Percentage inhibitory activity	[54]
Antimicrobial Activity	The paper disc diffusion method employed to determine the antimicrobial activity of the essential oils. For the assays, cultures of the following microorganisms are used: two Gram positive (*S. aureus* and *S. epidermidis*) and two Gram-negative (*Pseudomonas aeruginosa* and *E. coli*) Bacteria, and two yeasts (*Saccharomyces cerevisiae* and *Candida albicans*). Suspensions of the tested microorganisms are spread onto solid media plates. Filter paper discs are individually impregnated with 50 ml essential oil then lay onto the surfaces of the inoculated plates. At the end of the incubation time (24 h at 37 °C for bacteria, 48 h at 25 °C for yeasts), positive antibacterial and	Width (mm, including the diameter of the disc) of the zone of inhibition after incubation	[39]

	antifungal activities established by the presence of measurable zones of inhibition.		
Total Flavonoids Content	An aliquot of diluted sample solution mixed with distilled water and 5 % $NaNO_2$ solution. After 6 min, 10 % $AlCl_3$ solution added and allowed to stand for few min, then 4 % NaOH solution added to the mixture. Immediately, water is added to bring the final volume to 5 mL, and then the mixture is thoroughly mixed and allowed to stand for another 15 min and absorbance taken at 510 nm. Rutin is used as standard compound for the quantification of total flavonoids.	mg of rutin equiv. per 100 gram of fresh peel	[55]
Ash content determination	1–2 g of the sample accurately weighed into a weighed empty crucible separately. The crucible placed in a furnace and heated for 3–4 h at 600 °C to burn off all the organic matter. The crucible is taken out of the furnace and placed in a desiccator to cool and weighed. $$\text{Ash content (\%)} = \frac{\text{Weight of ash}}{\text{weight of sample}} \times 100$$	Percent of ash	[56]
Equivalent weight determination	Weighed pectin sample and transferred into a 250 mL conical flask and ethanol, NaCl added to it. Later, distilled water and few drops of phenol red indicator are added to the mixture. The solution slowly titrated (to avoid possible deesterification) with 0.1 M NaOH to endpoint of pink color. $$\text{Equivalent weight} = \frac{\text{weight of pectin sample}}{\text{ml of alkali} \times \text{Normality of alkali}} \times 1000$$	Equivalent to NaOH molarity	[56]
Methoxyl content determination	To the neutral solution titrated for equivalent weight, containing pectic substances, 0.25 N NaOH is added and shaken thoroughly; allowed to stand	Percentage of methoxyl content	[56]

	for 30 min at room temperature in a stoppered flask; after that 0.25 N HCl is added. The contents are titrated with 0.1 N NaOH until pink color as end point. $\text{Methoxyl content (\%)} = \frac{\text{ml of alkali} \times \text{normality of alkali} \times 3.1}{\text{weight ofsample}}$		
Moisture content determination	A dried empty petri dish dried in an oven, cooled in a desiccator and weighed. Five grams of the pectin samples transferred into the crucibles in the oven and heated at 130 °C for 1 h. The petri dish cooled to room temperature in a desiccator and weighed. $\text{Moisture content (\%)} = \frac{\text{Wt. of the pectin sample after drying}}{\text{Wt. of pectin sample}} \times 100$	Percentage of water content	[56]
Alkalinity assay Anhydrounic acid	To determine the alkalinity of ash, the ash is dissolved in 25 mL of 0.1 N HCl. The contents are heated and cooled to room temperature. This mixture is titrated with 0.1 N NaOH using phenolphthalein indicator until end point of orange color. $\text{Alkalinity (\%) as carbonate} = \frac{\text{Volume of NaOH} \times 60 \times 60}{\text{Wt. of ash} \times 1000}$ $\text{Anhydrounic acid} = \frac{\text{m. e. alkali for free acid} \times \text{m. e. alkali for saponofication} \times \text{m. e. titrable ash}}{\text{Wt. of sample (mg)}}$ where, m.e.= mili equivalent	Percentage of carbonate	[56]
**Galacturonic acid (GA), sugars and ethanol contents**			
	10 mL of sample centrifuged at 4000 rpm and 4 °C for 8 min, and the supernatant filtered to determine sugars, GA, and ethanol. The sugars	In percent	[57]

	(glucose, fructose, galactose, arabinose, sucrose, rhamnose, and xylose) and galacturonic acid (GA) are analyzed by ionic chromatography. Ethanol is quantified by injecting 0.8 L of filtered supernatant into a gas chromatograph with FID detector. The ethanol standard curve was determined for concentrations between 0.02 and 5% (*v*/*v*).		
GA	Galacturonic acid is determined by m-hydroxydiphenyl method. Samples mixed thoroughly with 0.125 M sodium tetraborate solution (in concentrated sulfuric acid) in an ice bath. The mixtures heated in a boiling bath for 5 min and subsequently cooled in an ice bath; the mixtures added with 0.15% m-hydroxydiphenyl (in 0.5 % NaOH) and mixed; A pink color develops during 5 min. After that, the absorbance recorded at 520 nm.	µg	[58]
Total sugars	By phenol–sulfuric acid method: Samples mixed thoroughly with aqueous solution of phenol of 5 %. Then concentrated sulfuric acid quickly introduced into the reaction medium. After homogenization, the mixtures heated in a boiling bath for 5 min, cooled in an ice bath and placed in the dark for 30 min. An orange color appears. The absorbance recorded at 492 nm. A standard curve was obtained using glucose at 25, 50, 100 and 200 µg $mL^{-1.}$	Microgram per milliliter	[58]
Ethanol analysis	Quantified in a gas chromatography	Volume by volume	[59,60]
Degree of esterification (DE)	Pectin dissolved in ethanol, 1 g NaCl and some drops of phenolphthalein. The solution is titrated with 0.1 N NaOH, V1; then NaOH was added in this solution which stirred at room temperature for 30 minutes. After that, 0.25 N	Percentage	[61] [58]

	HCl is added and the solutions shaken until the pink color disappeared. The solution is titrated again with 0.1 N NaOH, V2; DE value is calculated according to the following formula below: $$\%\ \mathrm{DE} = \frac{\mathrm{V2} \times 100}{\mathrm{V1} + \mathrm{V2}}$$		
Total dry matter content	Citrus pulp pellets obtained after fermentation and filtration, used to determine total dry matter by drying at 70 °C for 20 h, followed by drying in a vacuum oven at 70 °C for 1 h.	Weight	[60]
Para-anisidine value (PAV)	The sample dissolved in n-hexane, and the absorbance of the mixture measured at 350 nm (A1). Para-anisidine reagent (1mL) added to 5 mL of the mixture and held in the dark for 10 min before absorbance reading (A2) at 350 nm. The result is calculated as $PAV = 25\ (1.2A_2 - A_1)/m$, Where, m represents mass of sample oil.	$Gram^{-1}$	[51]
d-Limonene analysis	Scott method: based on a bromination reaction with the double bonds of the molecule. For most flavor and specialty chemical applications, *d*-limonene is analyzed instrumentally by GC/MS.	ml	[62]
Pectin content	Crude pectin added in 250 ml flask, then adding 0.1N NaOH and soaked for 7 hours, then added 1 N CH_3COOH and $CaCl_2$ after 5 minutes and kept it for 1 hour; the solution is boiled, filtered and dried; Calcium pectate is washed with hot water until not having Cl^- ion in the solution, dried at 105°C. The pure level of pectin is calculated according to the following formula below:	Percentage	[61]

$$P = \frac{m \times 0.92 \times 100}{M}$$

P (%): the pure level of pectin

m (g): weight of calcium pectate

M (g): weight of crude pectin

0.92: pectins have 92% in volume of calcium pectate

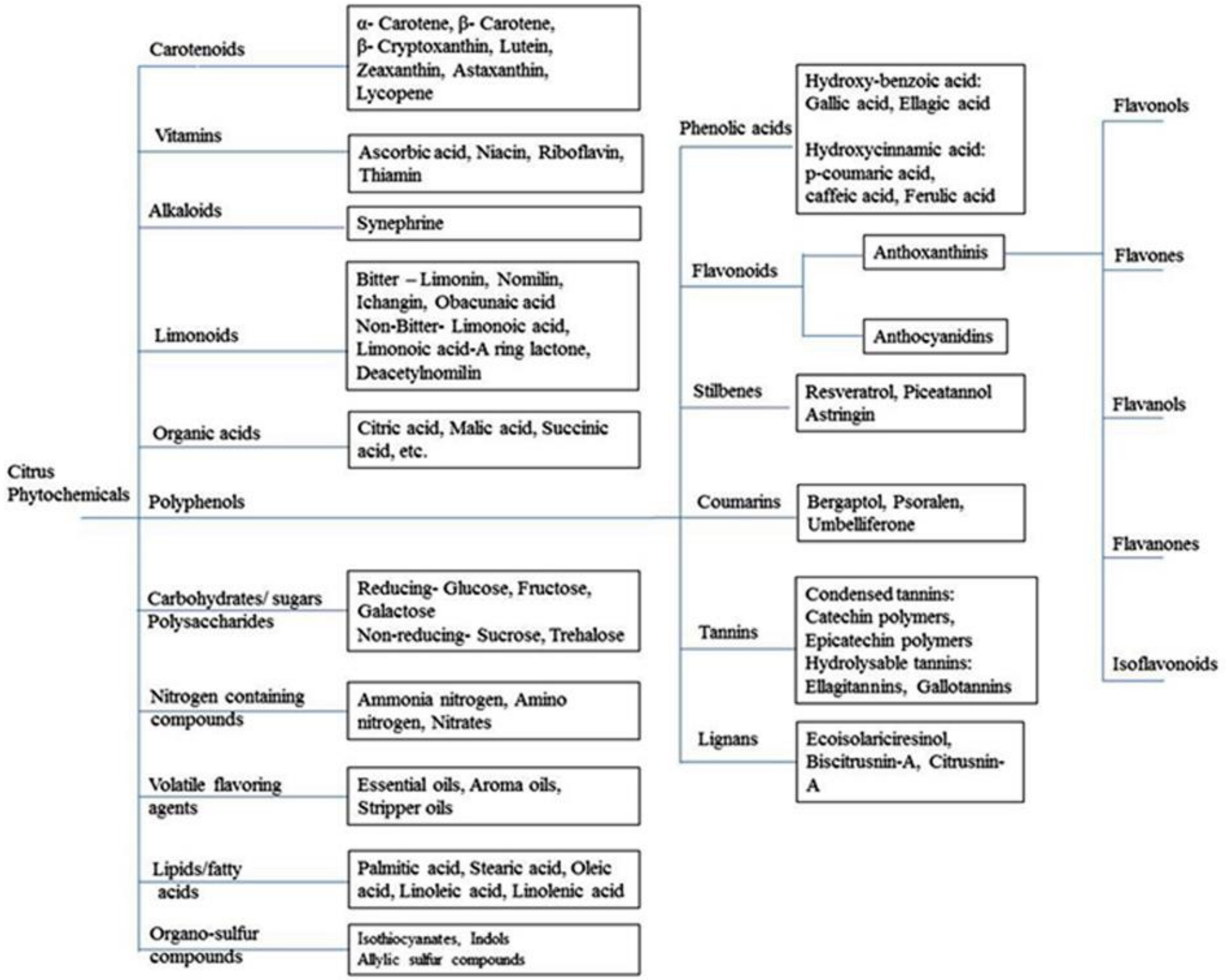

Figure S-5: Classification of major citrus phytochemicals extracted from different parts of citrus wastes

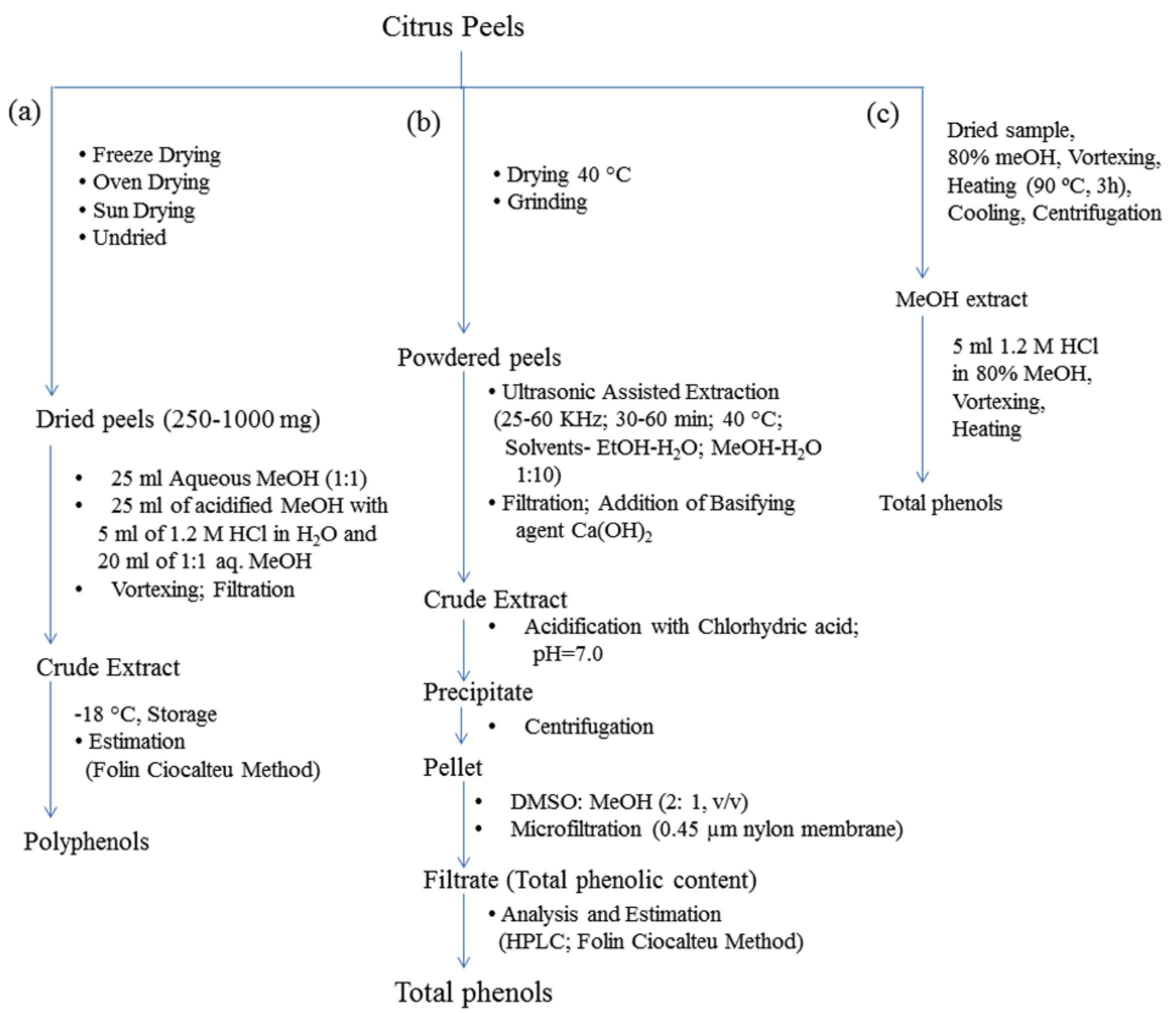

Figure S-6: Steps involved in the different extraction method employed for total polyphenolic content from citrus peels [63-65]

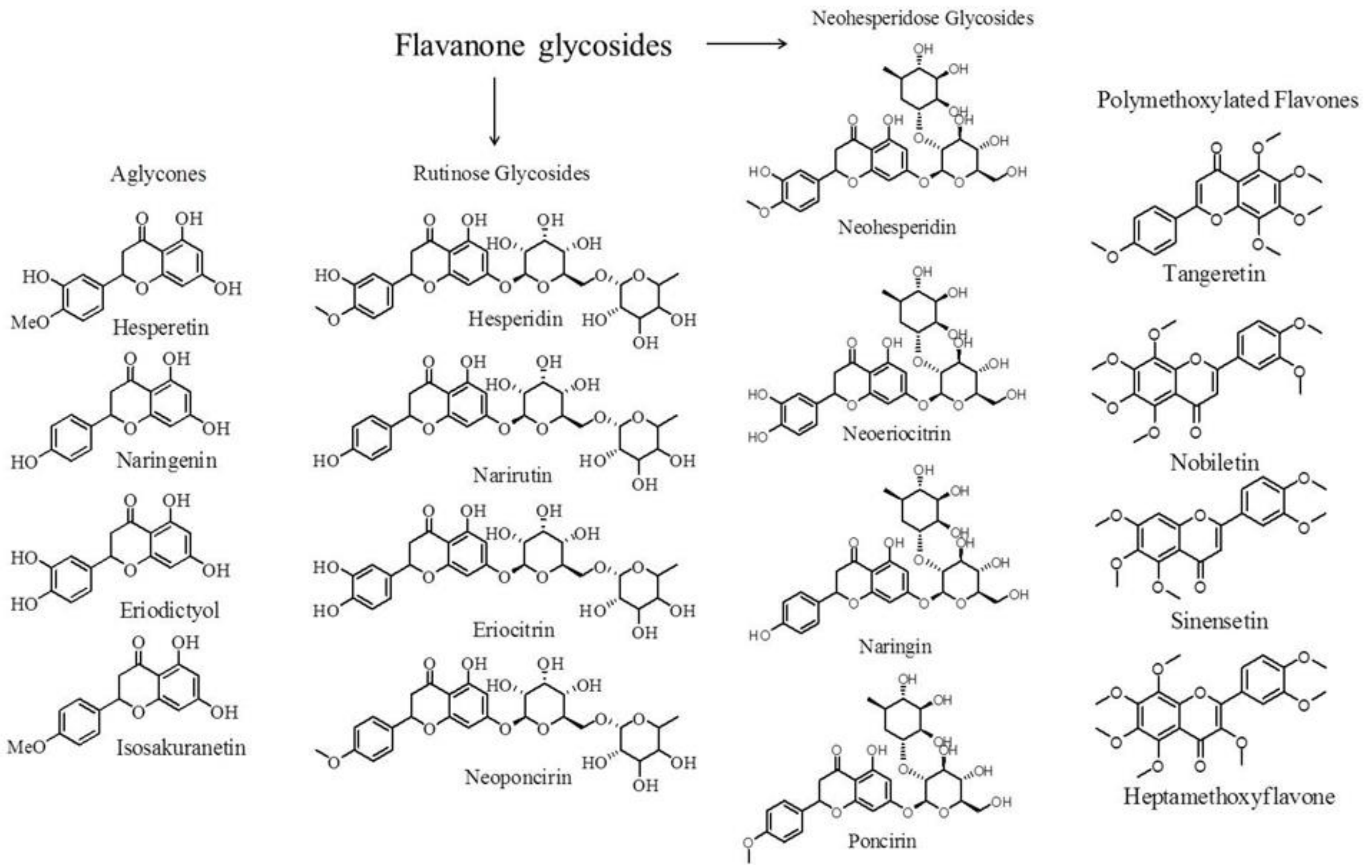

Figure S-7: Molecular structures of major flavonoids: aglycones, glucosides, and polymethoxylated forms

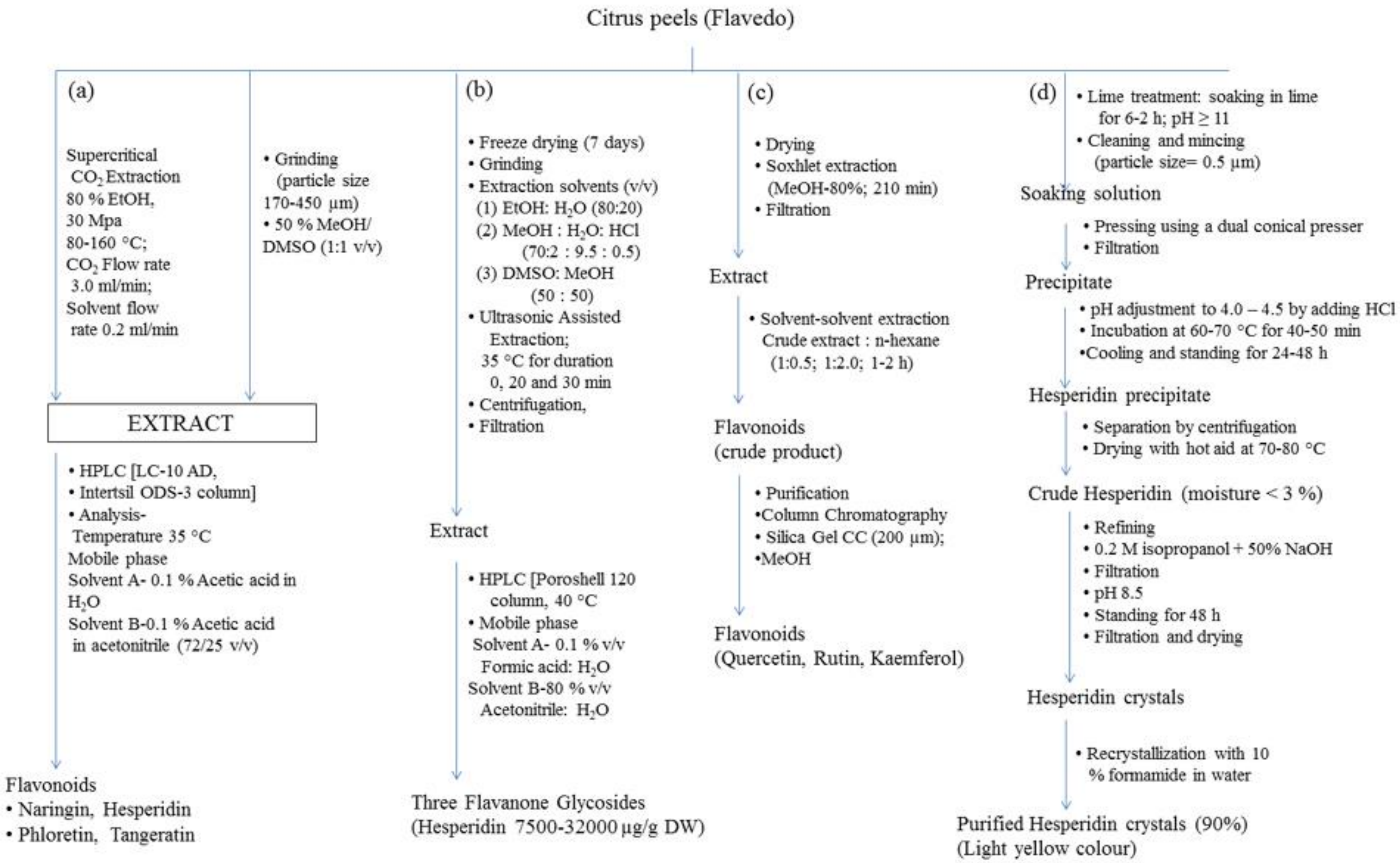
Citrus peels (Flavedo)
(a)
Supercritical CO2 Extraction
80 % EtOH,
30 Mpa
80-160 °C;
CO2 Flow rate 3.0 ml/min;
Solvent flow rate 0.2 ml/min
• Grinding (particle size 170-450 µm)
• 50 % MeOH/ DMSO (1:1 v/v)
EXTRACT
• HPLC [LC-10 AD,
• Intertsil ODS-3 column]
• Analysis-
Temperature 35 °C
Mobile phase
Solvent A- 0.1 % Acetic acid in H2O
Solvent B-0.1 % Acetic acid in acetonitrile (72/25 v/v)
Flavonoids
• Naringin, Hesperidin
• Phloretin, Tangeratin
(b)
• Freeze drying (7 days)
• Grinding
• Extraction solvents (v/v)
(1) EtOH: H2O (80:20)
(2) MeOH : H2O: HCl (70:2 : 9.5 : 0.5)
(3) DMSO: MeOH (50 : 50)
• Ultrasonic Assisted Extraction;
35 °C for duration 0, 20 and 30 min
• Centrifugation,
• Filtration
Extract
• HPLC [Poroshell 120 column, 40 °C
• Mobile phase
Solvent A- 0.1 % v/v Formic acid: H2O
Solvent B-80 % v/v Acetonitrile: H2O
Three Flavanone Glycosides
(Hesperidin 7500-32000 µg/g DW)
(c)
• Drying
• Soxhlet extraction (MeOH-80%; 210 min)
• Filtration
Extract
• Solvent-solvent extraction
Crude extract : n-hexane (1:0.5; 1:2.0; 1-2 h)
Flavonoids
(crude product)
• Purification
•Column Chromatography
• Silica Gel CC (200 µm);
•MeOH
Flavonoids
(Quercetin, Rutin, Kaemferol)
(d)
• Lime treatment: soaking in lime for 6-2 h; pH ≥ 11
• Cleaning and mincing (particle size= 0.5 µm)
Soaking solution
• Pressing using a dual conical presser
• Filtration
Precipitate
• pH adjustment to 4.0 – 4.5 by adding HCl
• Incubation at 60-70 °C for 40-50 min
•Cooling and standing for 24-48 h
Hesperidin precipitate
• Separation by centrifugation
• Drying with hot aid at 70-80 °C
Crude Hesperidin (moisture < 3 %)
• Refining
• 0.2 M isopropanol + 50% NaOH
• Filtration
• pH 8.5
• Standing for 48 h
• Filtration and drying
Hesperidin crystals
• Recrystallization with 10 % formamide in water
Purified Hesperidin crystals (90%)
(Light yellow colour)

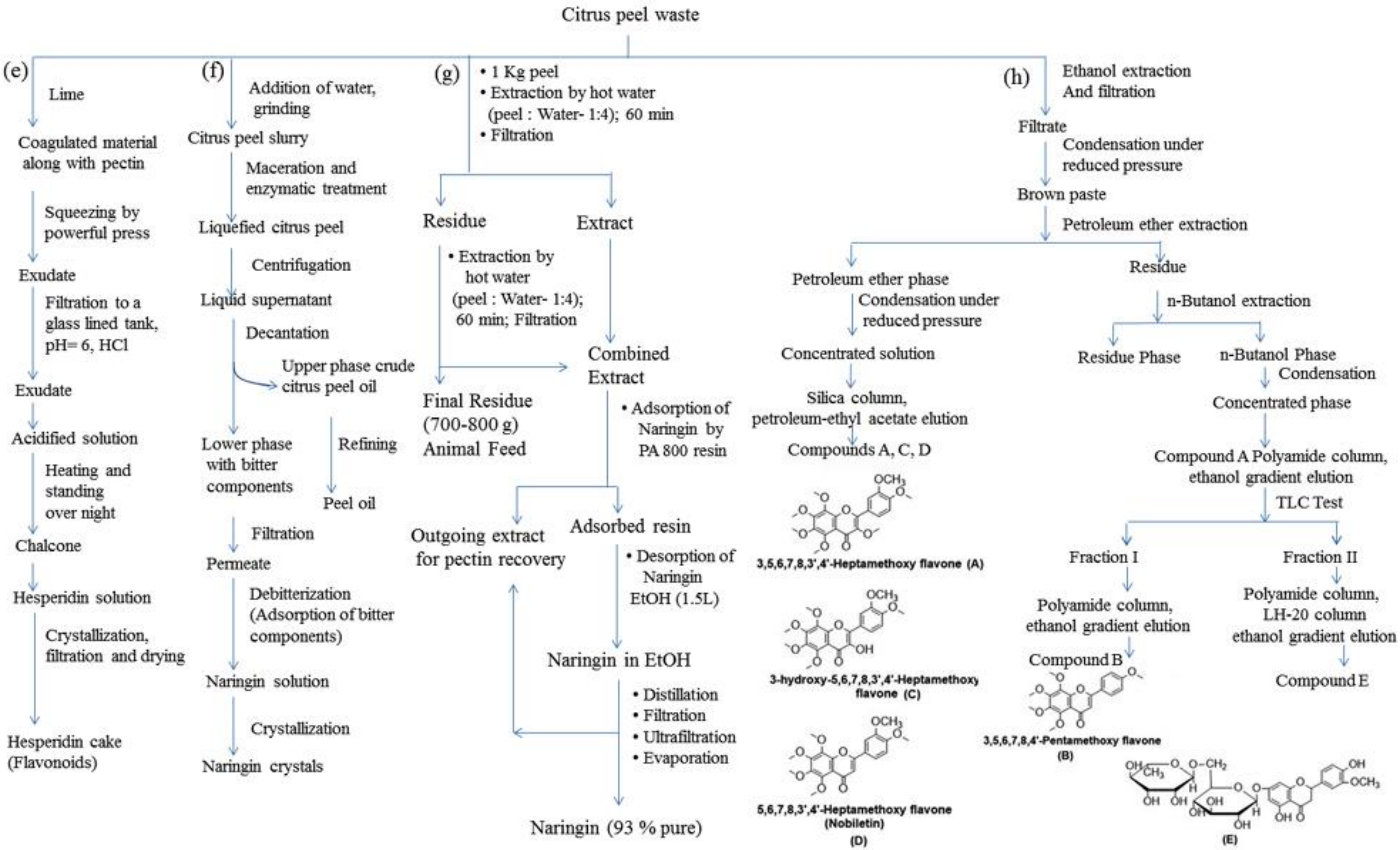
Citrus peel waste
(e)
Lime
Coagulated material along with pectin
Squeezing by powerful press
Exudate
Filtration to a glass lined tank, pH= 6, HCl
Exudate
Acidified solution
Heating and standing over night
Chalcone
Hesperidin solution
Crystallization, filtration and drying
Hesperidin cake (Flavonoids)
(f)
Addition of water, grinding
Citrus peel slurry
Maceration and enzymatic treatment
Liquefied citrus peel
Centrifugation
Liquid supernatant
Decantation
Upper phase crude citrus peel oil
Refining
Peel oil
Lower phase with bitter components
Filtration
Permeate
Debitterization (Adsorption of bitter components)
Naringin solution
Crystallization
Naringin crystals
(g)
• 1 Kg peel
• Extraction by hot water (peel : Water- 1:4); 60 min
• Filtration
Residue
Extract
• Extraction by hot water (peel : Water- 1:4); 60 min; Filtration
Combined Extract
Final Residue (700-800 g) Animal Feed
• Adsorption of Naringin by PA 800 resin
Outgoing extract for pectin recovery
Adsorbed resin
• Desorption of Naringin EtOH (1.5L)
Naringin in EtOH
• Distillation
• Filtration
• Ultrafiltration
• Evaporation
Naringin (93 % pure)
(h)
Ethanol extraction And filtration
Filtrate
Condensation under reduced pressure
Brown paste
Petroleum ether extraction
Petroleum ether phase
Condensation under reduced pressure
Concentrated solution
Silica column, petroleum-ethyl acetate elution
Compounds A, C, D
3,5,6,7,8,3',4'-Heptamethoxy flavone (A)
3-hydroxy-5,6,7,8,3',4'-Heptamethoxy flavone (C)
5,6,7,8,3',4'-Heptamethoxy flavone (Nobiletin) (D)
Residue
n-Butanol extraction
Residue Phase
n-Butanol Phase
Condensation
Concentrated phase
Compound A Polyamide column, ethanol gradient elution
TLC Test
Fraction I
Fraction II
Polyamide column, ethanol gradient elution
Polyamide column, LH-20 column ethanol gradient elution
Compound B
Compound E
3,5,6,7,8,4'-Pentamethoxy flavone (B)
(E)

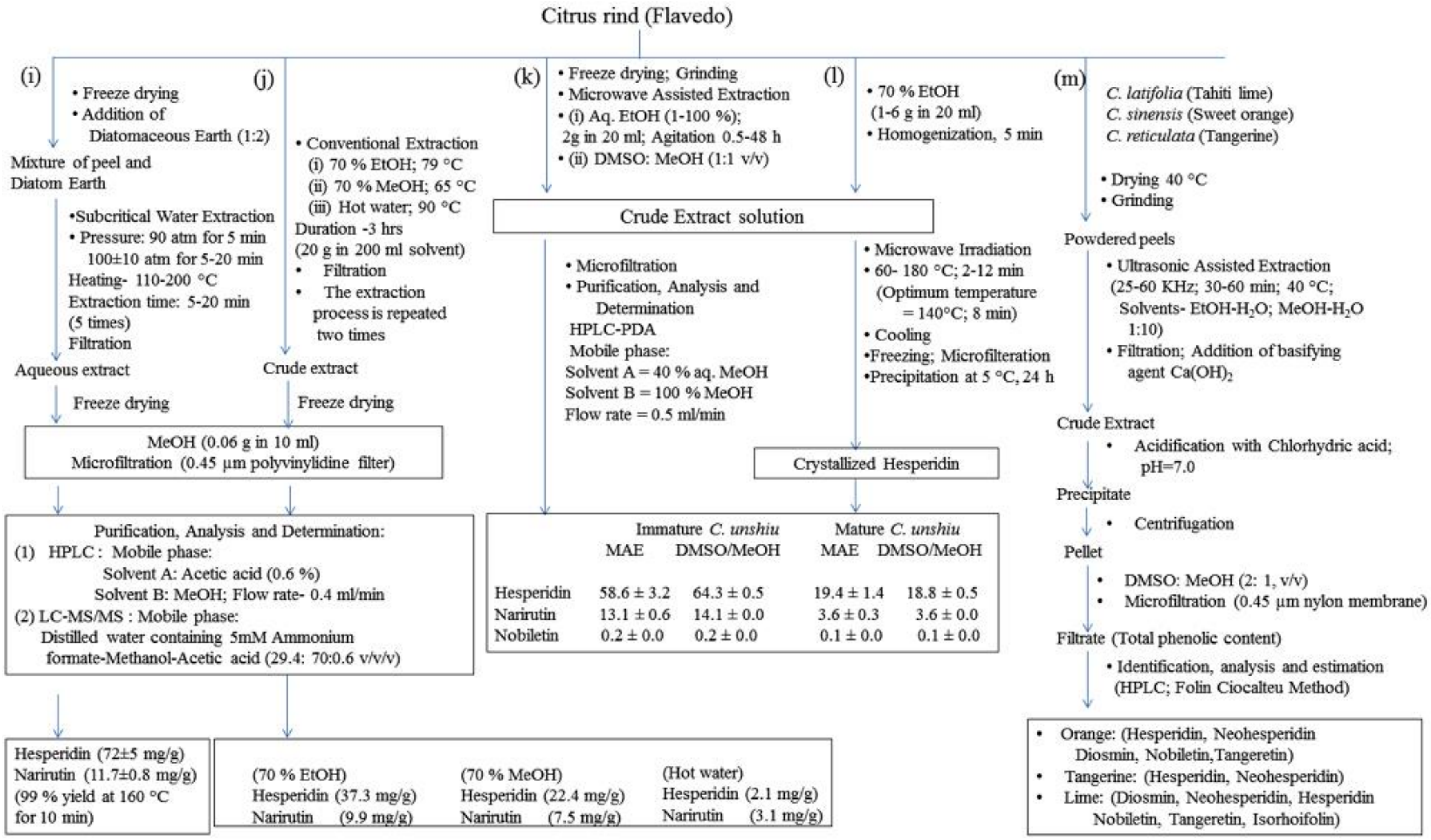

Figure S-8: Steps involved in the extraction and purification of flavonoids from citrus peels [64, 66-74]

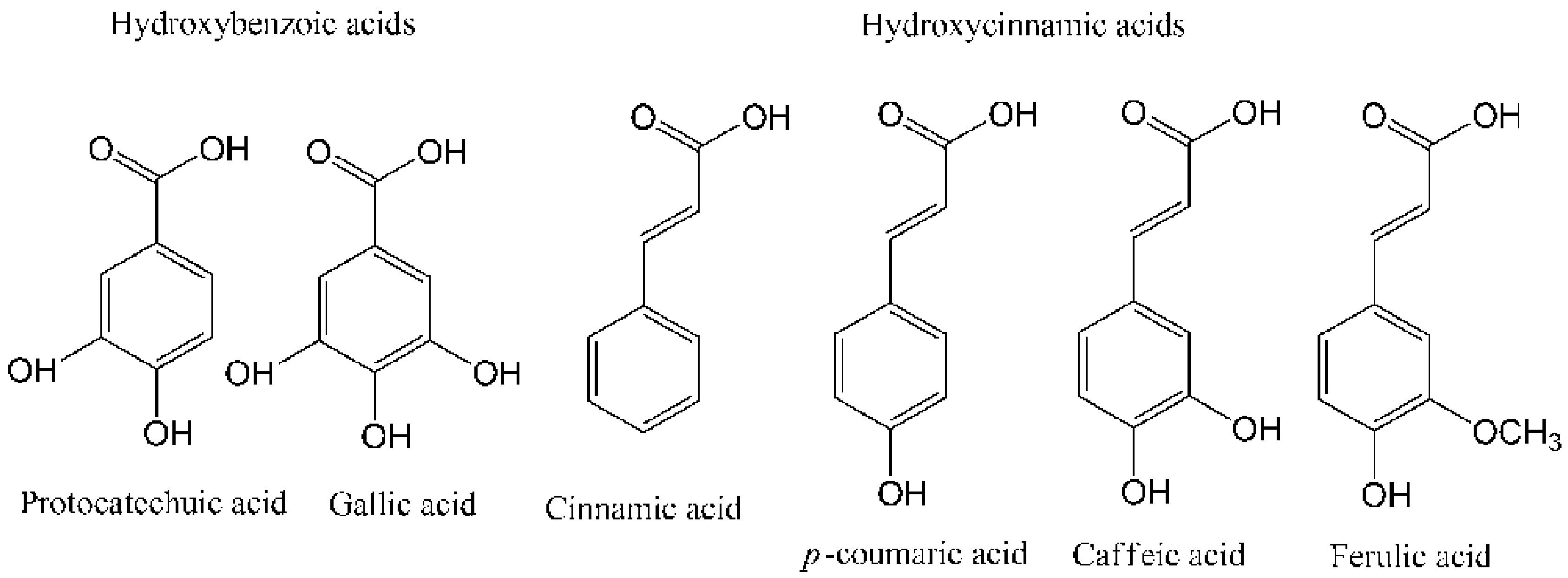

Figure S-9: Molecular structures of phenolic acids found in citrus fruits

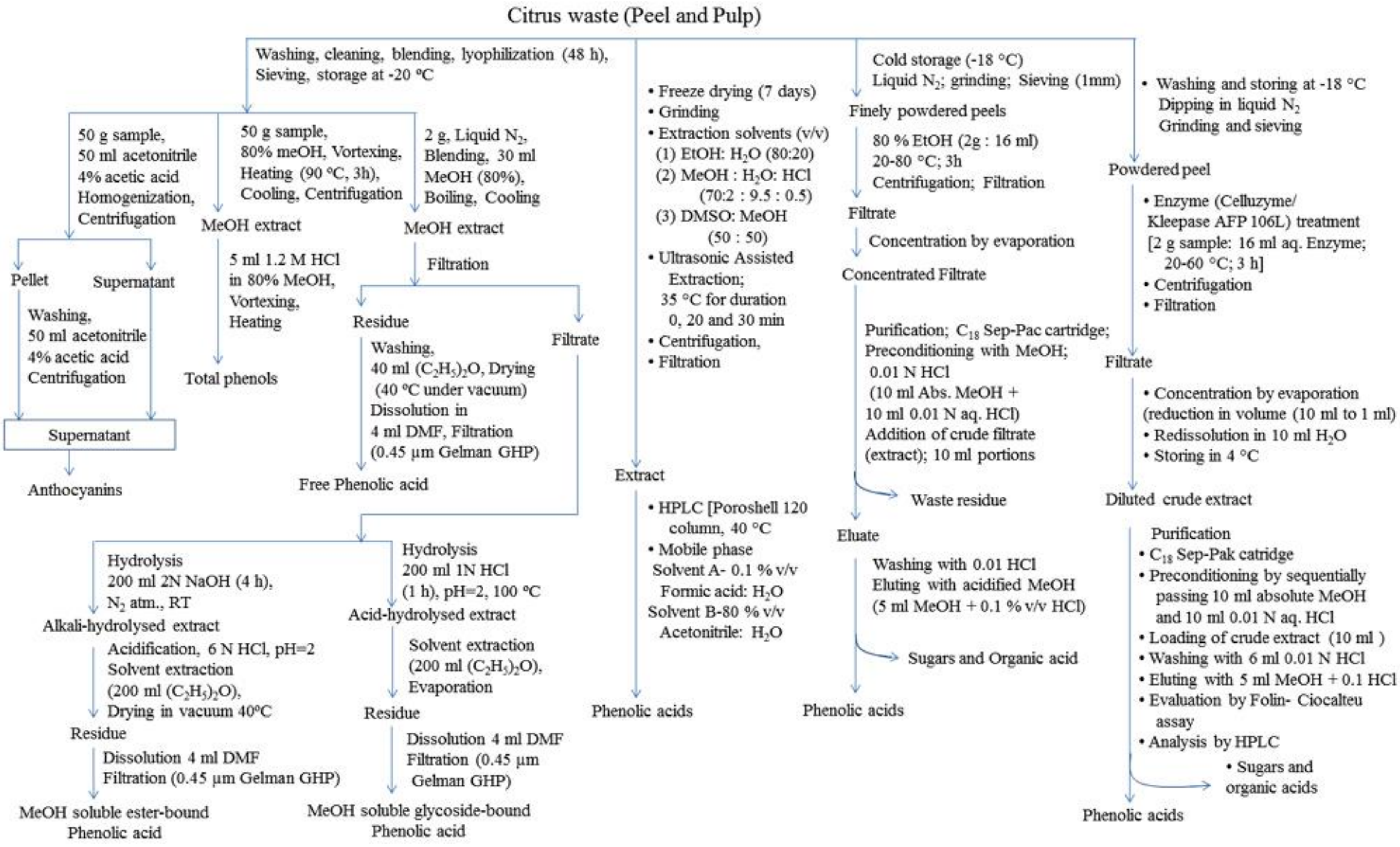

Figure S-10: Steps involved in the extraction of total phenols, anthocyanins and phenolic acids from citrus waste [47, 75-77]

(a) Limonoid aglycones

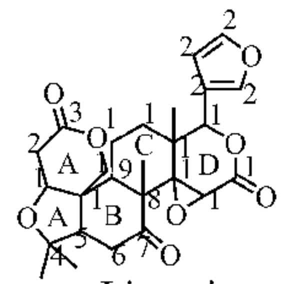

Limoni

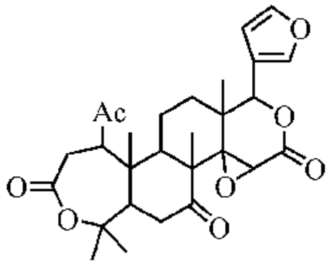

Nomili

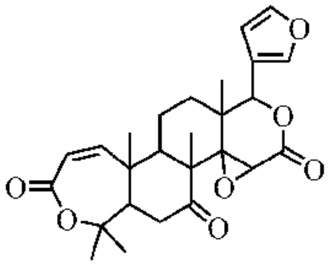

Obacunon

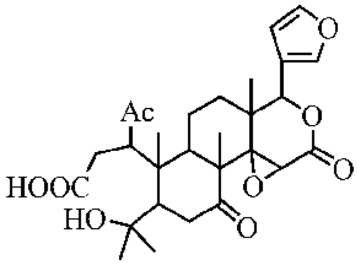

Nomilinic

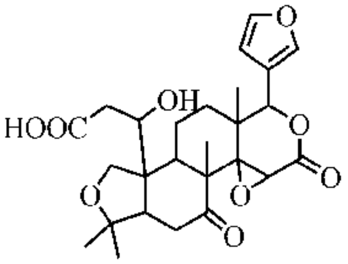

Isolimonic

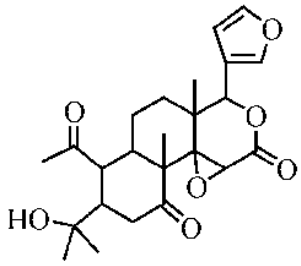

Ichangensi

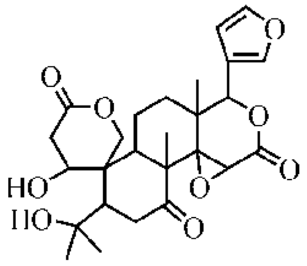

Ichangi

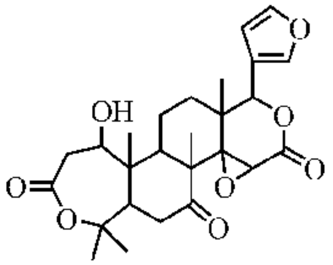

Deacetylnomili

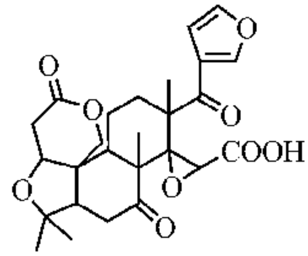

17-Dehydrolimonoate A-ring

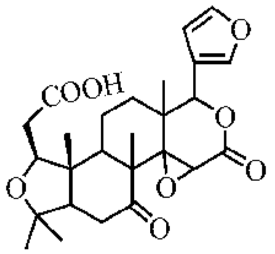

Isoobacunoic acid

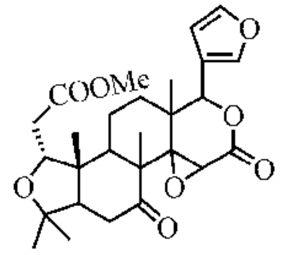

Methyl Isoobacunoate

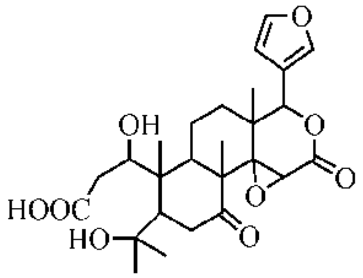

Deacetylnomilinic

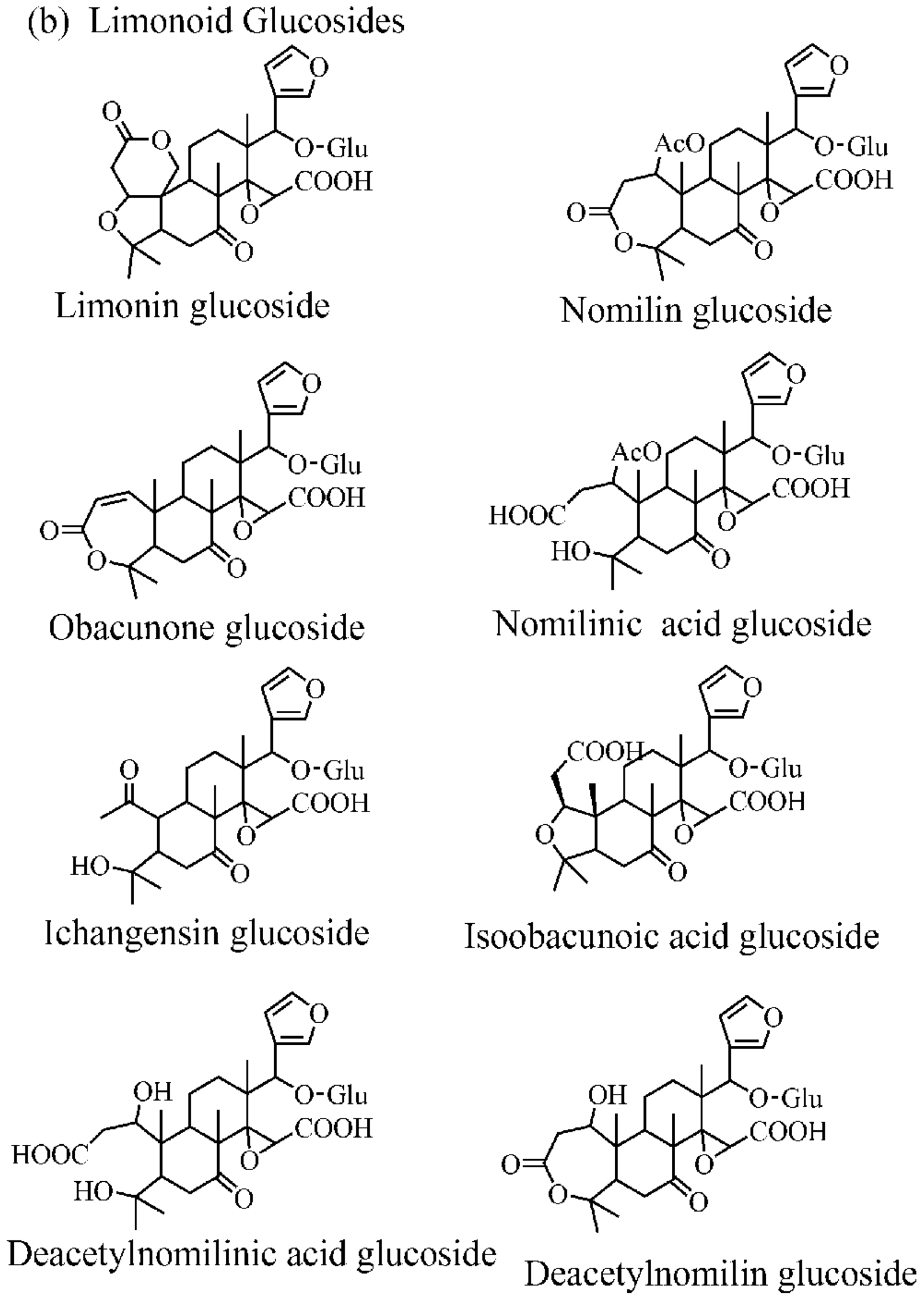

Figure S-11: Molecular structures of common citrus (a) limonoid aglycones and (b) limonoid glucosides [78]

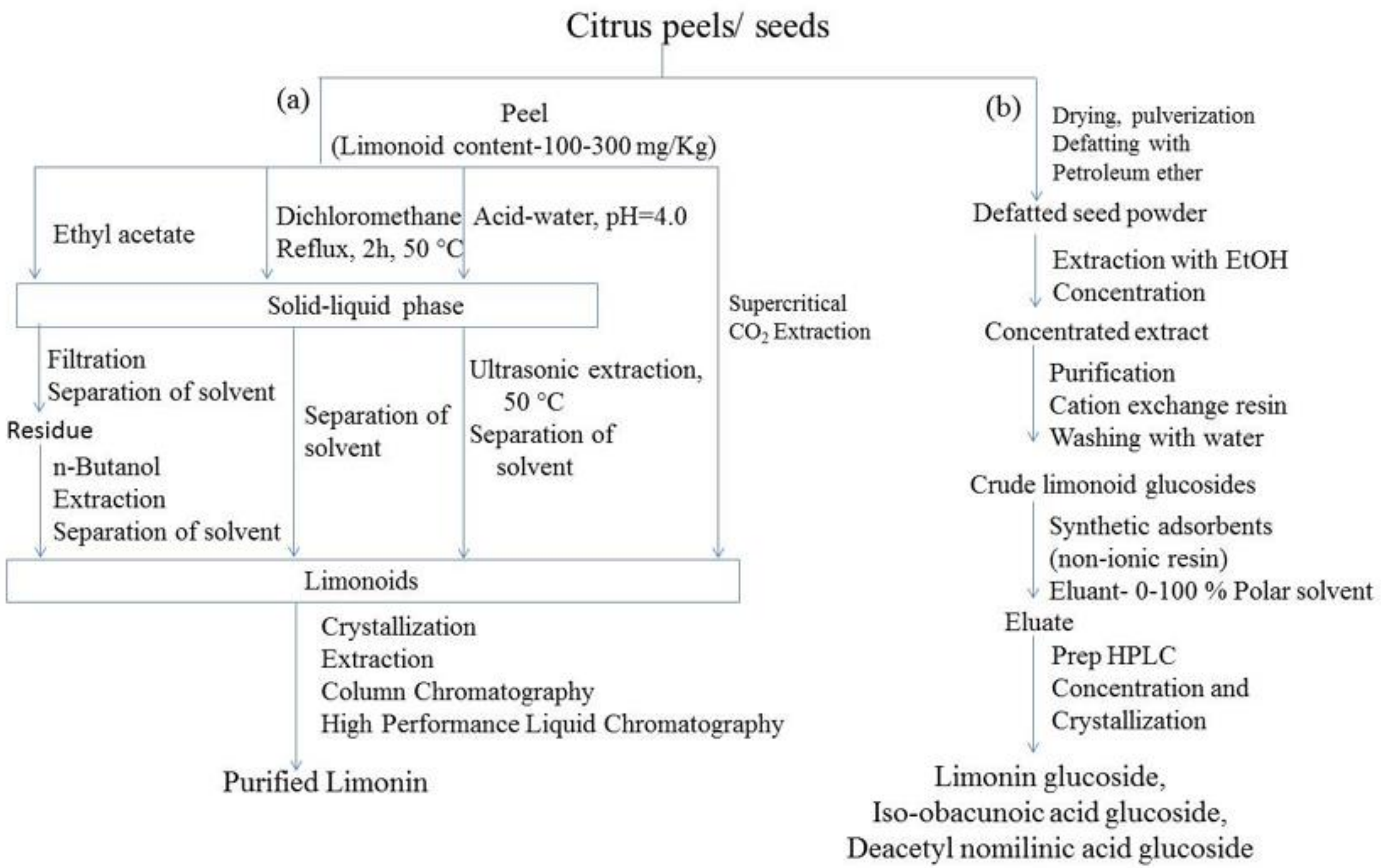
Citrus peels/ seeds
(a)
Peel
(Limonoid content-100-300 mg/Kg)
Ethyl acetate
Dichloromethane
Reflux, 2h, 50 °C
Acid-water, pH=4.0
Solid-liquid phase
Supercritical
CO_2 Extraction
Filtration
Separation of solvent
Residue
n-Butanol
Extraction
Separation of solvent
Separation of
solvent
Ultrasonic extraction,
50 °C
Separation of
solvent
Limonoids
Crystallization
Extraction
Column Chromatography
High Performance Liquid Chromatography
Purified Limonin
(b)
Drying, pulverization
Defatting with
Petroleum ether
Defatted seed powder
Extraction with EtOH
Concentration
Concentrated extract
Purification
Cation exchange resin
Washing with water
Crude limonoid glucosides
Synthetic adsorbents
(non-ionic resin)
Eluant- 0-100 % Polar solvent
Eluate
Prep HPLC
Concentration and
Crystallization
Limonin glucoside,
Iso-obacunoic acid glucoside,
Deacetyl nomilinic acid glucoside

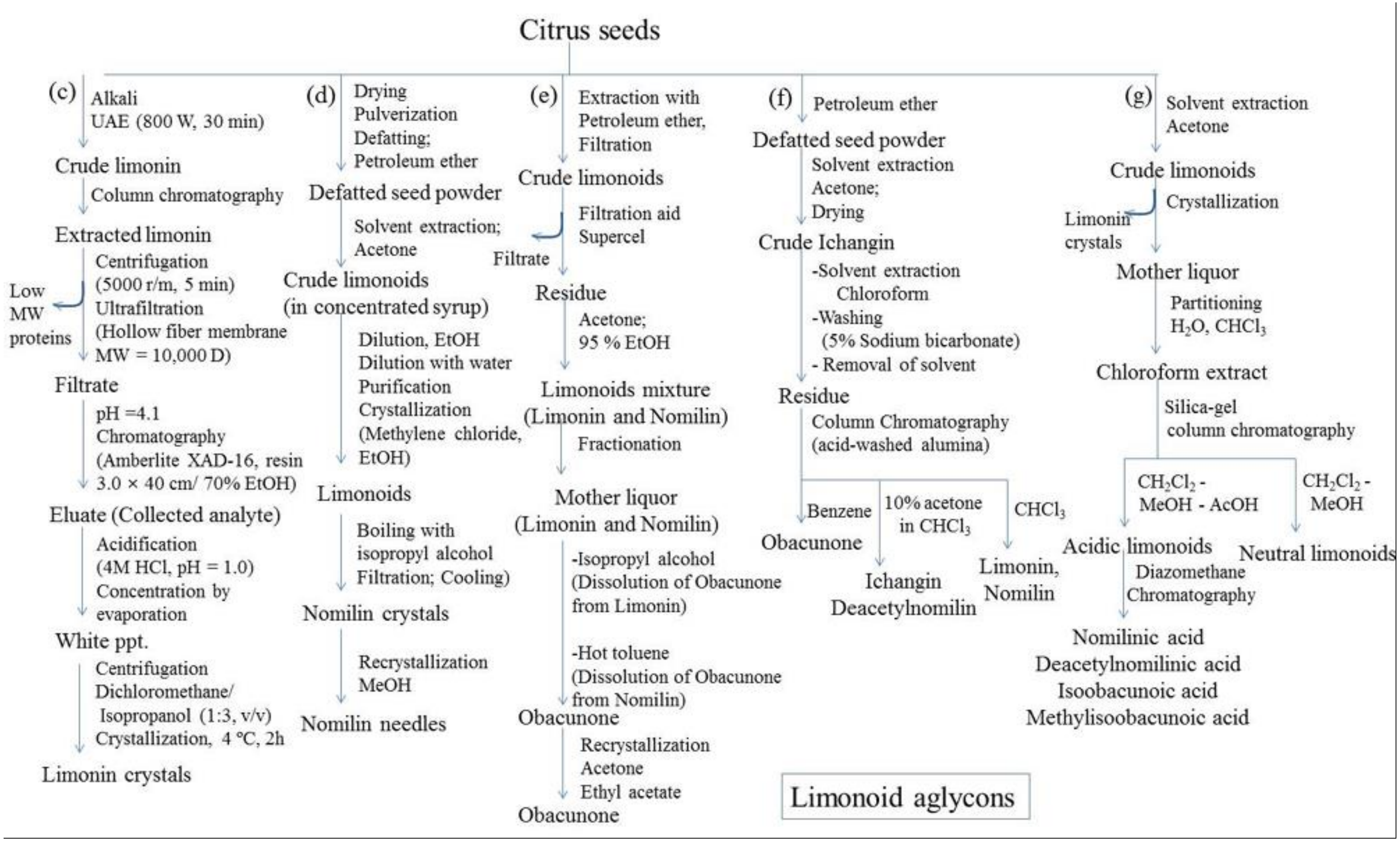

Citrus seeds
(c) Alkali
UAE (800 W, 30 min)
Crude limonin
Column chromatography
Extracted limonin
Centrifugation
(5000 r/m, 5 min)
Ultrafiltration
(Hollow fiber membrane
MW = 10,000 D)
Low MW proteins
Filtrate
pH =4.1
Chromatography
(Amberlite XAD-16, resin
3.0 × 40 cm/ 70% EtOH)
Eluate (Collected analyte)
Acidification
(4M HCl, pH = 1.0)
Concentration by
evaporation
White ppt.
Centrifugation
Dichloromethane/
Isopropanol (1:3, v/v)
Crystallization, 4 °C, 2h
Limonin crystals
(d) Drying
Pulverization
Defatting;
Petroleum ether
Defatted seed powder
Solvent extraction;
Acetone
Crude limonoids
(in concentrated syrup)
Dilution, EtOH
Dilution with water
Purification
Crystallization
(Methylene chloride,
EtOH)
Limonoids
Boiling with
isopropyl alcohol
Filtration; Cooling)
Nomilin crystals
Recrystallization
MeOH
Nomilin needles
(e) Extraction with
Petroleum ether,
Filtration
Crude limonoids
Filtration aid
Supercel
Filtrate
Residue
Acetone;
95 % EtOH
Limonoids mixture
(Limonin and Nomilin)
Fractionation
Mother liquor
(Limonin and Nomilin)
-Isopropyl alcohol
(Dissolution of Obacunone
from Limonin)
-Hot toluene
(Dissolution of Obacunone
from Nomilin)
Obacunone
Recrystallization
Acetone
Ethyl acetate
Obacunone
(f) Petroleum ether
Defatted seed powder
Solvent extraction
Acetone;
Drying
Crude Ichangin
-Solvent extraction
Chloroform
-Washing
(5% Sodium bicarbonate)
- Removal of solvent
Residue
Column Chromatography
(acid-washed alumina)
Benzene
10% acetone
in $CHCl_3$
$CHCl_3$
Obacunone
Ichangin
Deacetylnomilin
Limonin,
Nomilin
(g) Solvent extraction
Acetone
Crude limonoids
Crystallization
Limonin
crystals
Mother liquor
Partitioning
H_2O, $CHCl_3$
Chloroform extract
Silica-gel
column chromatography
CH_2Cl_2 -
MeOH - AcOH
CH_2Cl_2 -
MeOH
Acidic limonoids
Neutral limonoids
Diazomethane
Chromatography
Nomilinic acid
Deacetylnomilinic acid
Isoobacunoic acid
Methylisoobacunoic acid
Limonoid aglycons

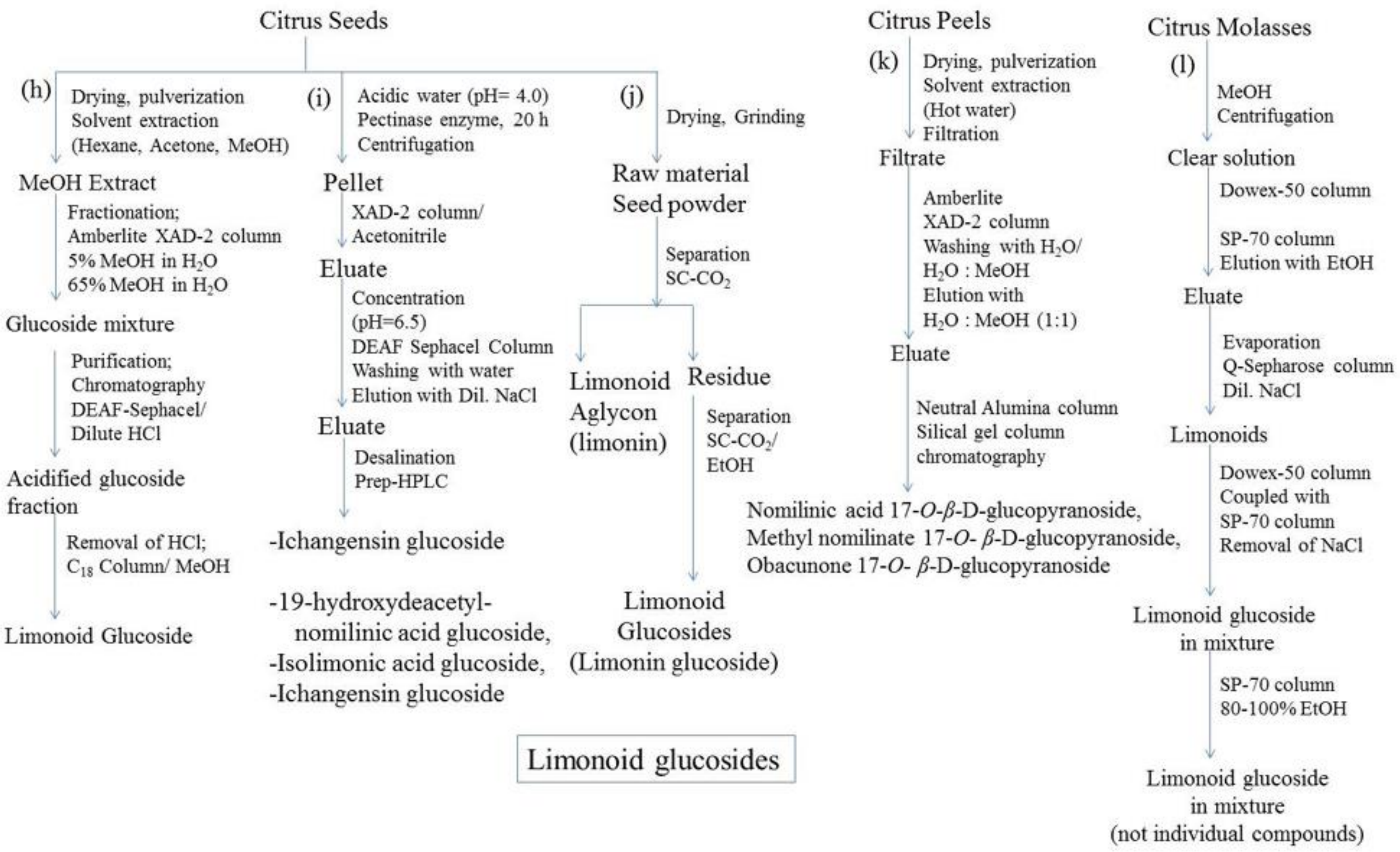

Figure S-12: (a-b) Steps involved in the different extraction methods for limonoids from citrus peels and seeds; (b-g) Limonoid aglycones, and (h-l) limonoid glucosides [79,80]

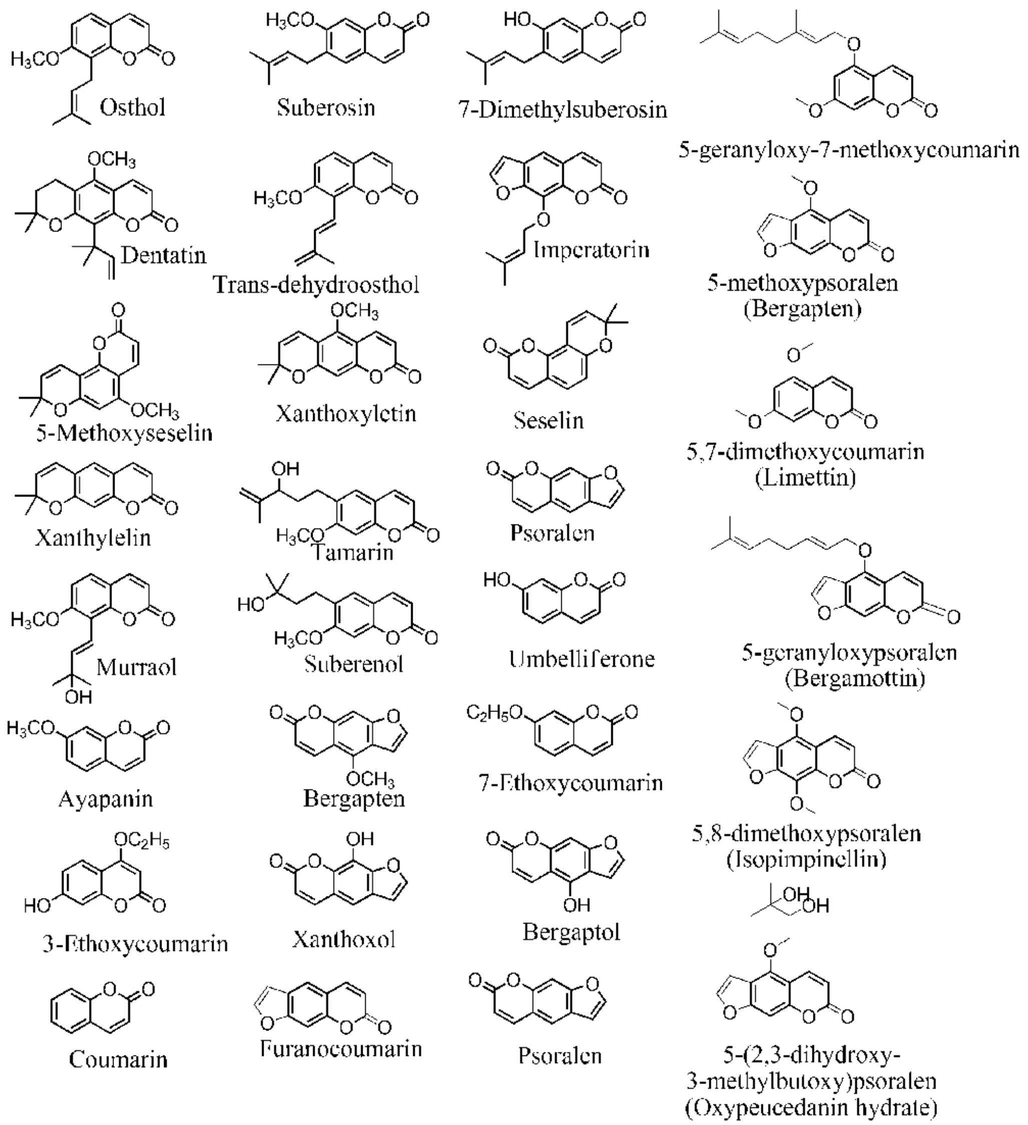

Figure S-13: Molecular structures of coumarins found in citrus wastes

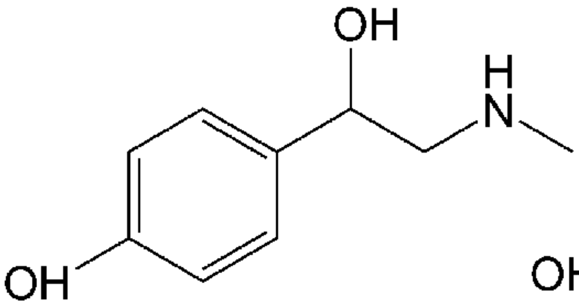

p-synaphrine

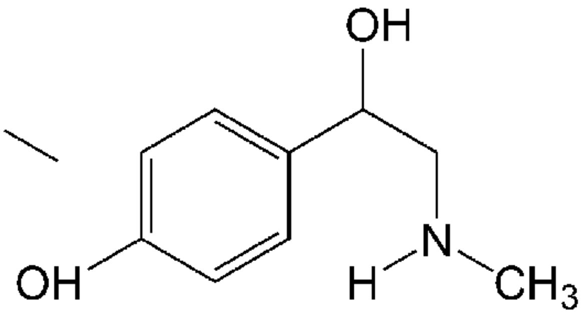

p-synaphrine

Figure S-14: Molecular structure of synaphrene and *p*- synaphrene

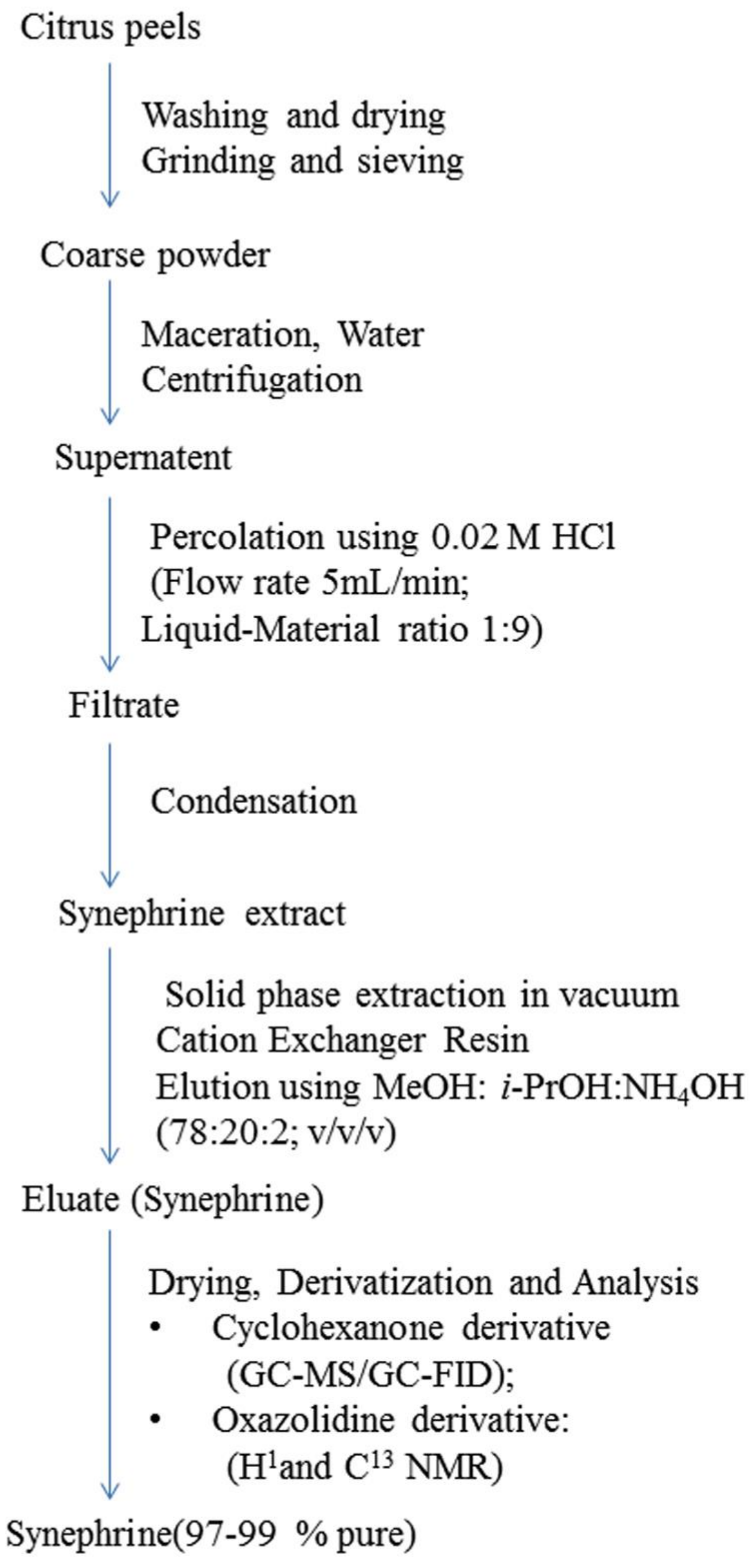

Figure S-15: Steps involved in the extraction of synaphrine [81]

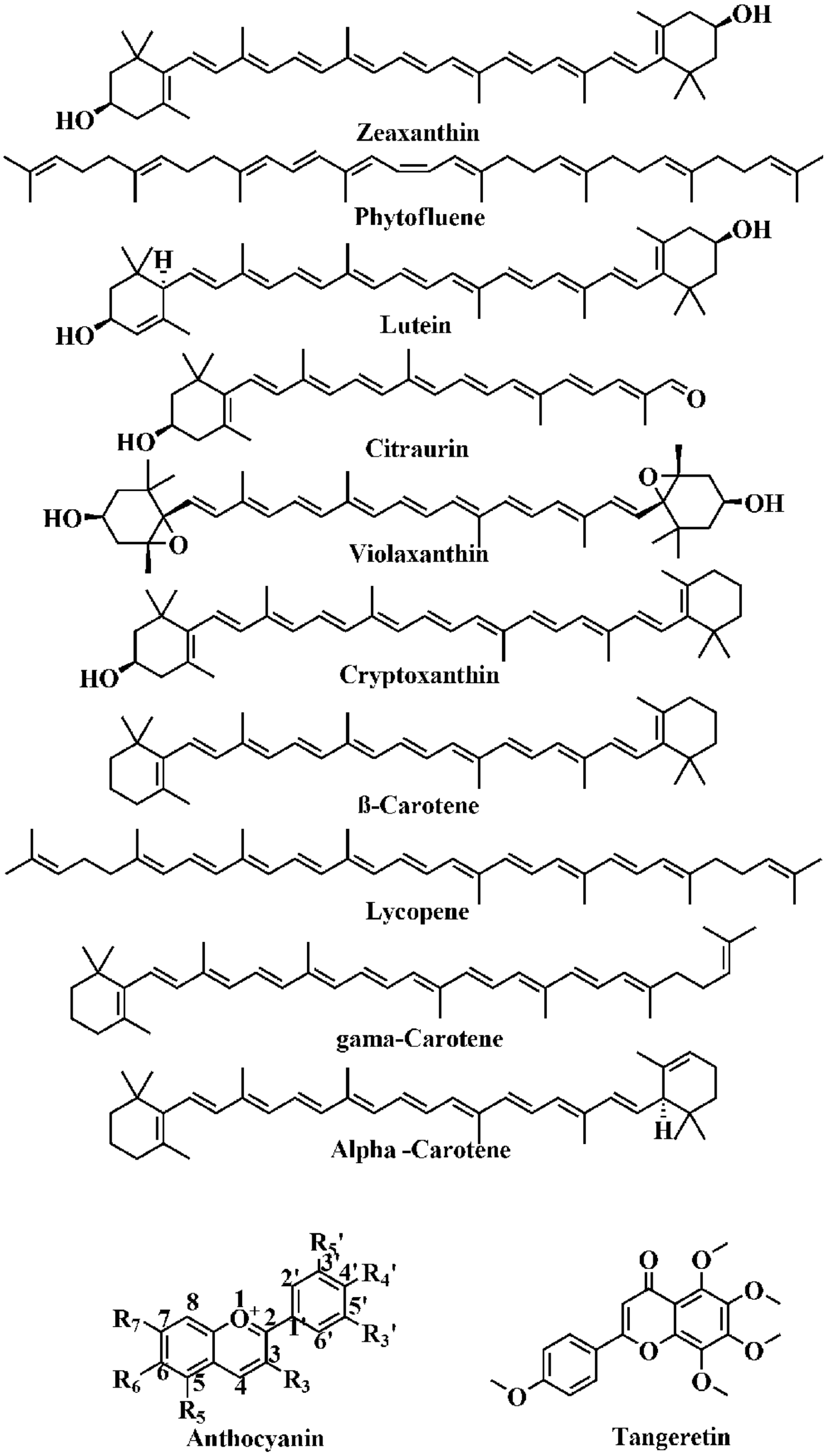

Figure S-16: Molecular structures of the pigments found in citrus wastes

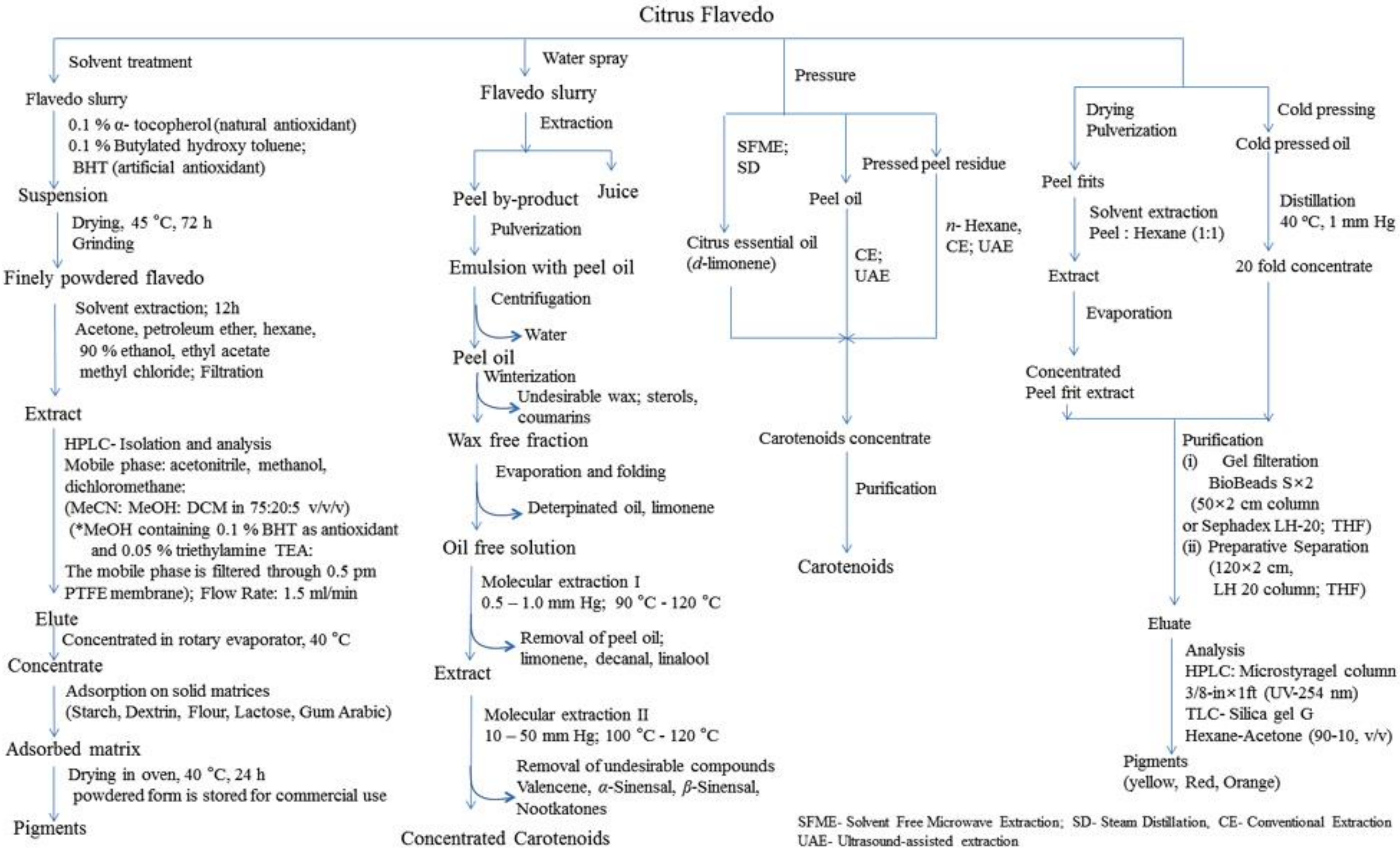

Figure S-17: Important steps in the extraction of carotenoids [82-85]

Table S-3: Composition of pigments in different citrus varieties [6]

	Valencia orange		Tangerine		Eureka Lemon		Ruby Red Grapefruit	
	Endocarp (15 mg/l)	Peels (120 mg/kg)	Endocarp (27 mg/l)	Peels (186 mg/kg)	Endocarp (0.6 mg/kg)	Peels (1.4 mg/kg)	Endocarp -	Peels -
(Approximate percentage of total carotenoids)								
Hydrocarbons								
Phytoene	4.0	3.1	5.8	4.2	-	-	1.6	47.0
Phytofluene	13.0	6.1	7.2	3.5	22.0	18.0	4.4	1.4
α-Carotene	0.5	0.1	0.3	0.2	6.6	6.8	-	0.1
β-Carotene	1.1	0.3	4.1	0.4	4.0	17.0	27.0	7.2
Mono-ols								
Cryptoxanthin	5.3	1.2	33.0	24.0	26.0	9.7	0.7	1.4
Cryptoflavin	0.5	1.2	0.8	3.4	-	-	-	1.3
3-Hydroxy- α-Carotene	1.5	0.3	1.0	0.6	-	-	0.2	0.1
Diols								
Lutein	2.9	1.2	2.9	3.3	-	-	0.3	0.9
Zeaxanthin	4.5	0.8	3.3	3.5	-	-	-	-
Monoetherdiols								
Antheraxanthin	5.8	6.3	9.7	6.2	-	-	0.7	-
Mutatoxanthin	6.2		2.2	2.8	-	-	0.4	0.2
Dietherdiols								
Violaxanthin	7.4	44.0	14	24.0	-	-	0.9	1.0
Luteoxanthin	17.0	16.0	3.5	9.1	-	-	0.4	1.8

Auroxanthin	12.0	2.3	0.4	1.9	-	-	0.3	1.6
Polyols								
Valenciaxanthin	2.8	2.2	0.2	0.4	-	-	-	-
Valenciachromes	1.0	0.7	-	-	-	-	0.2	0.3
Sinensiaxanthin	2.0	3.5	0.2	1.1	-	-	-	-

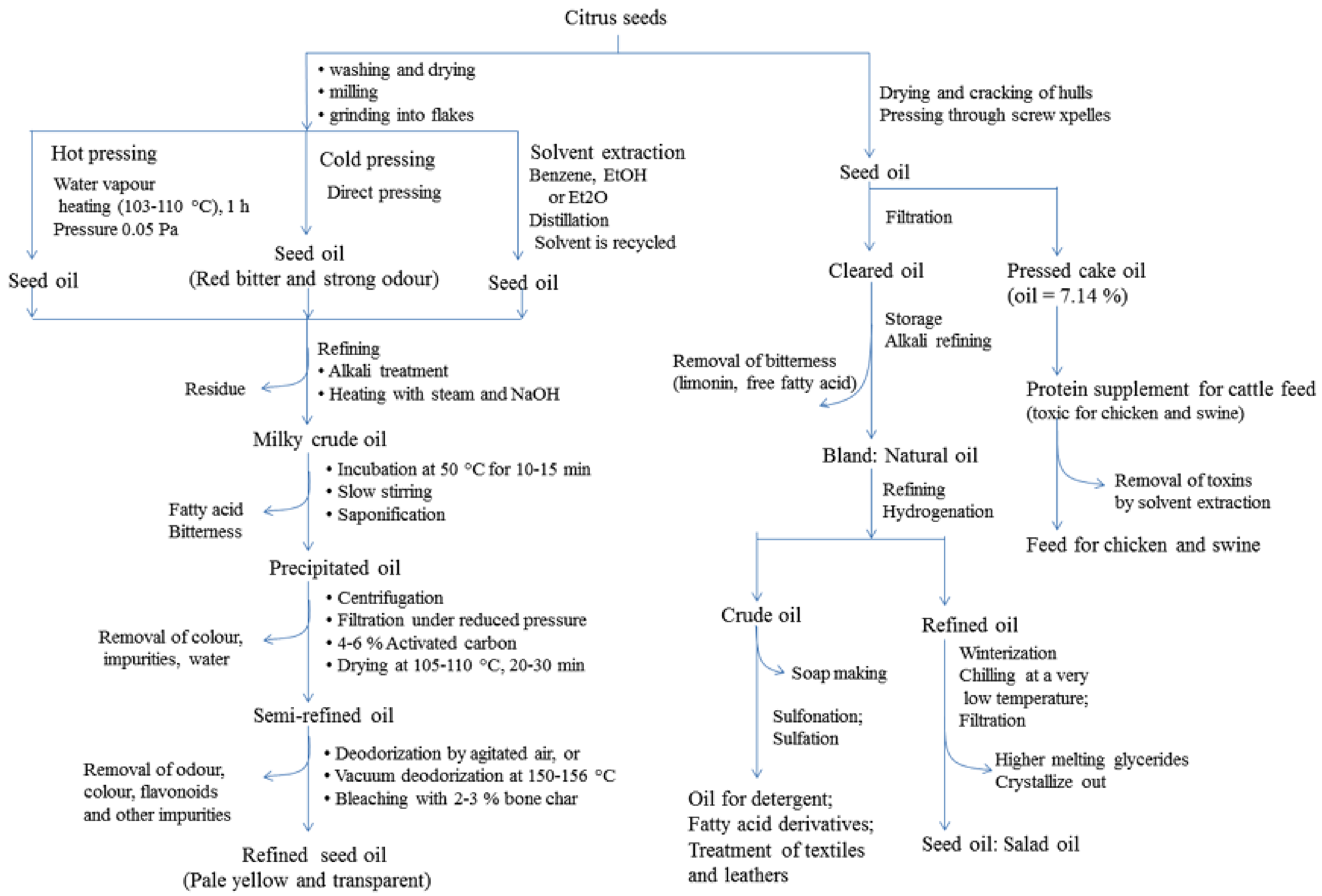

Figure S-18: Steps involved in the different extraction methods for seed oils from the citrus seeds [66,86]

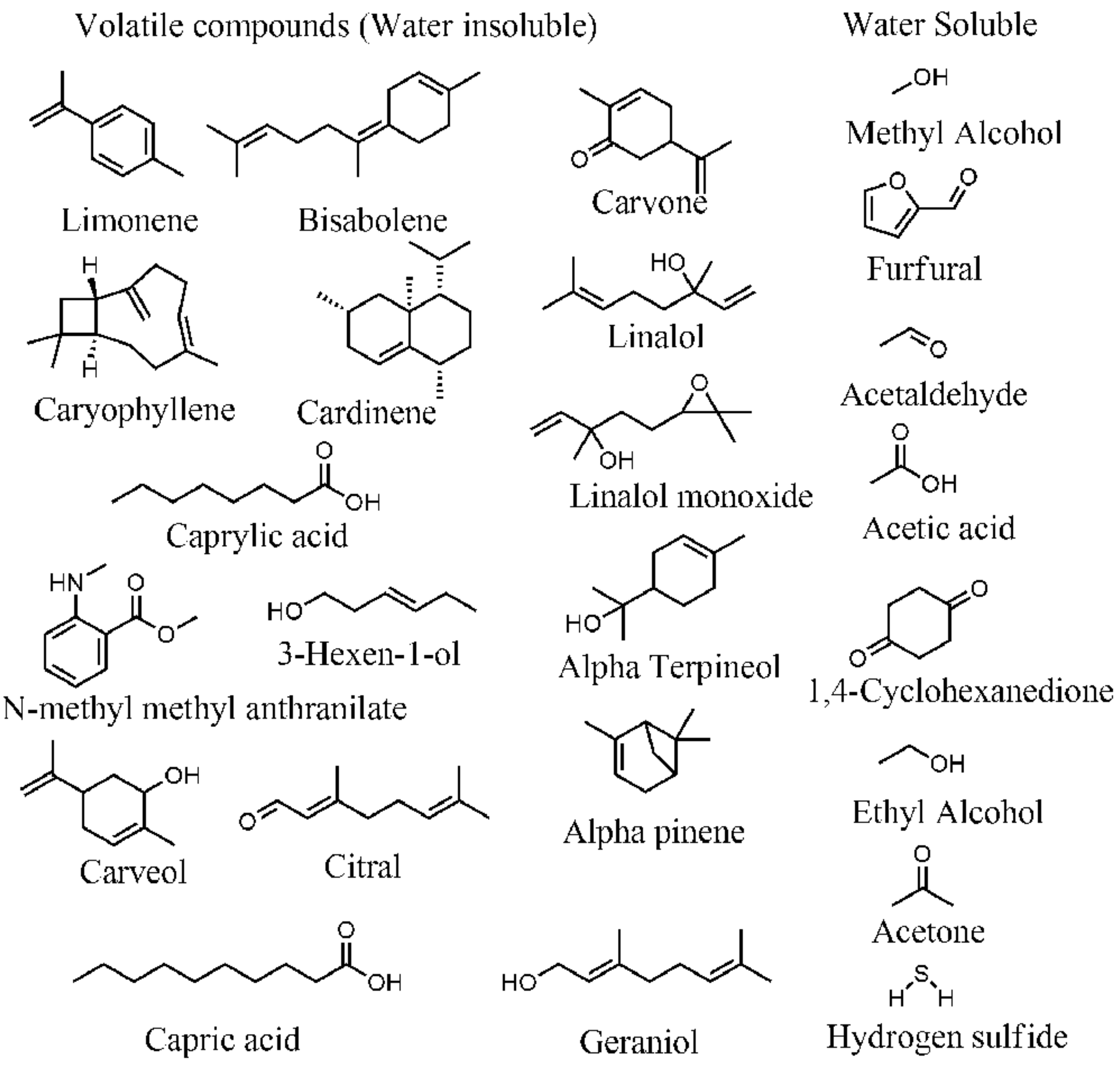

Figure S-19: Water soluble and insoluble volatile constituents found in citrus wastes

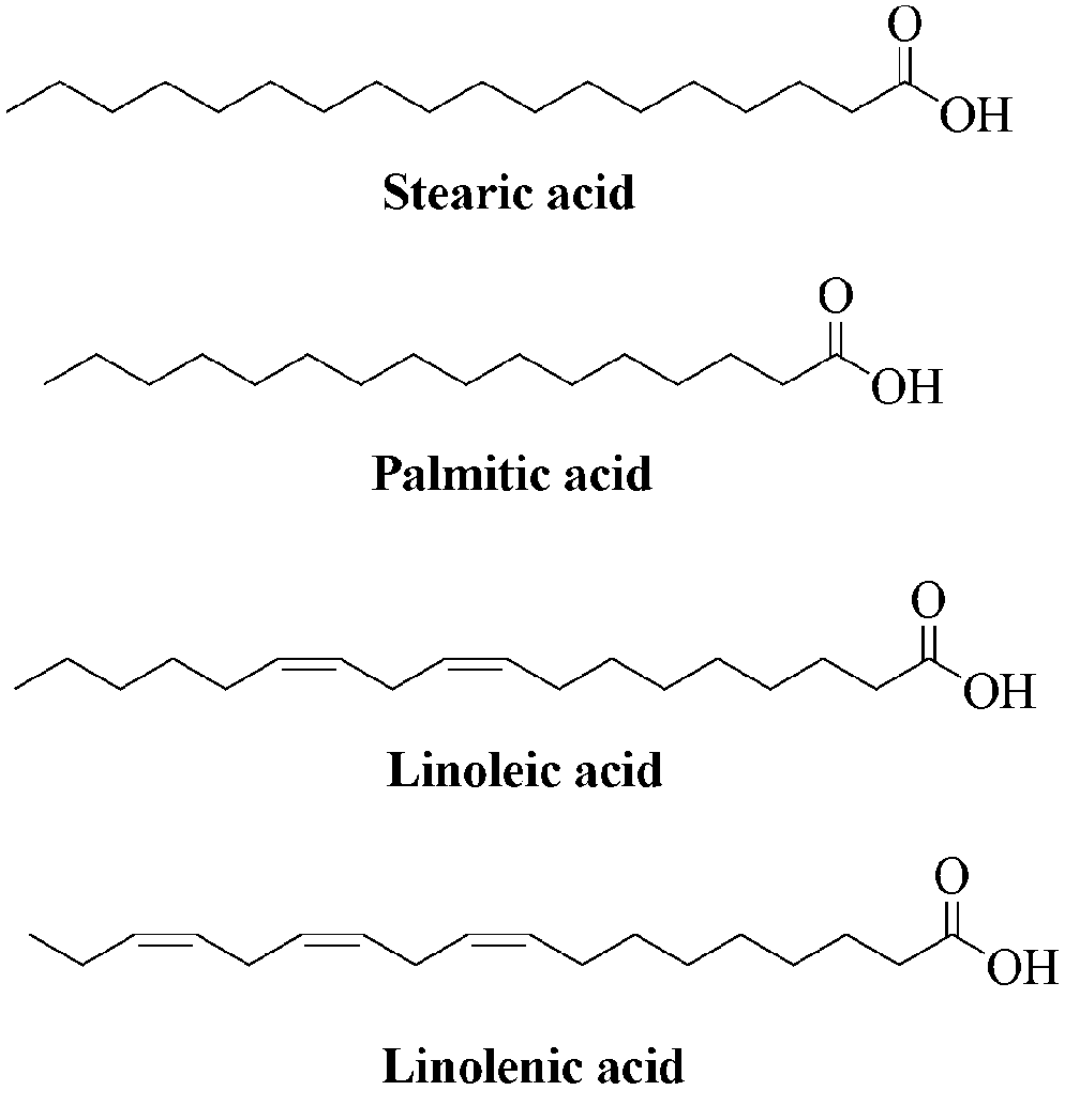

Figure S-20: Molecular structure of main lipids found in citrus wastes

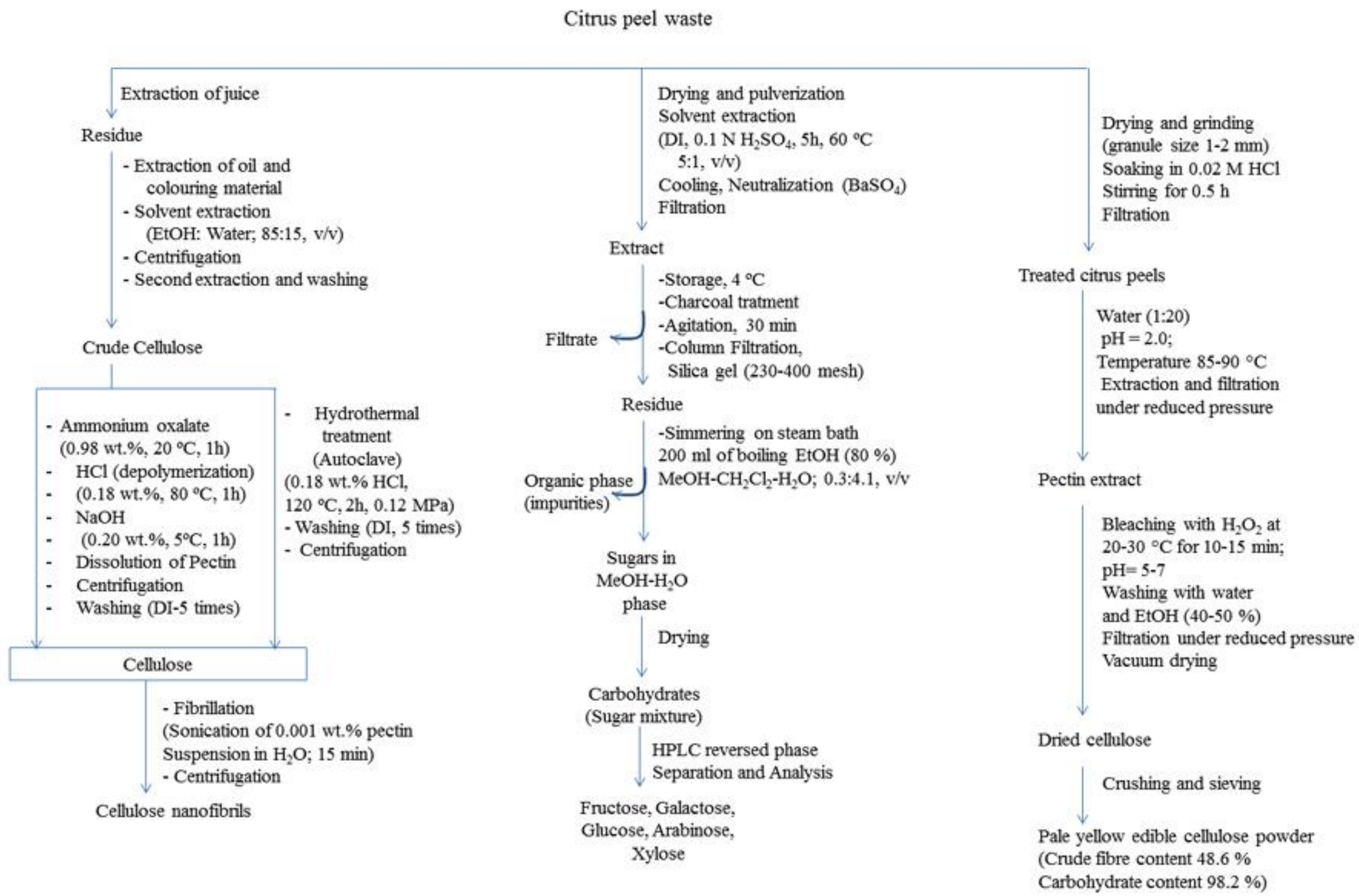

Figure S-21: Steps involved in the extraction of cellulose and sugars [66,87,88]

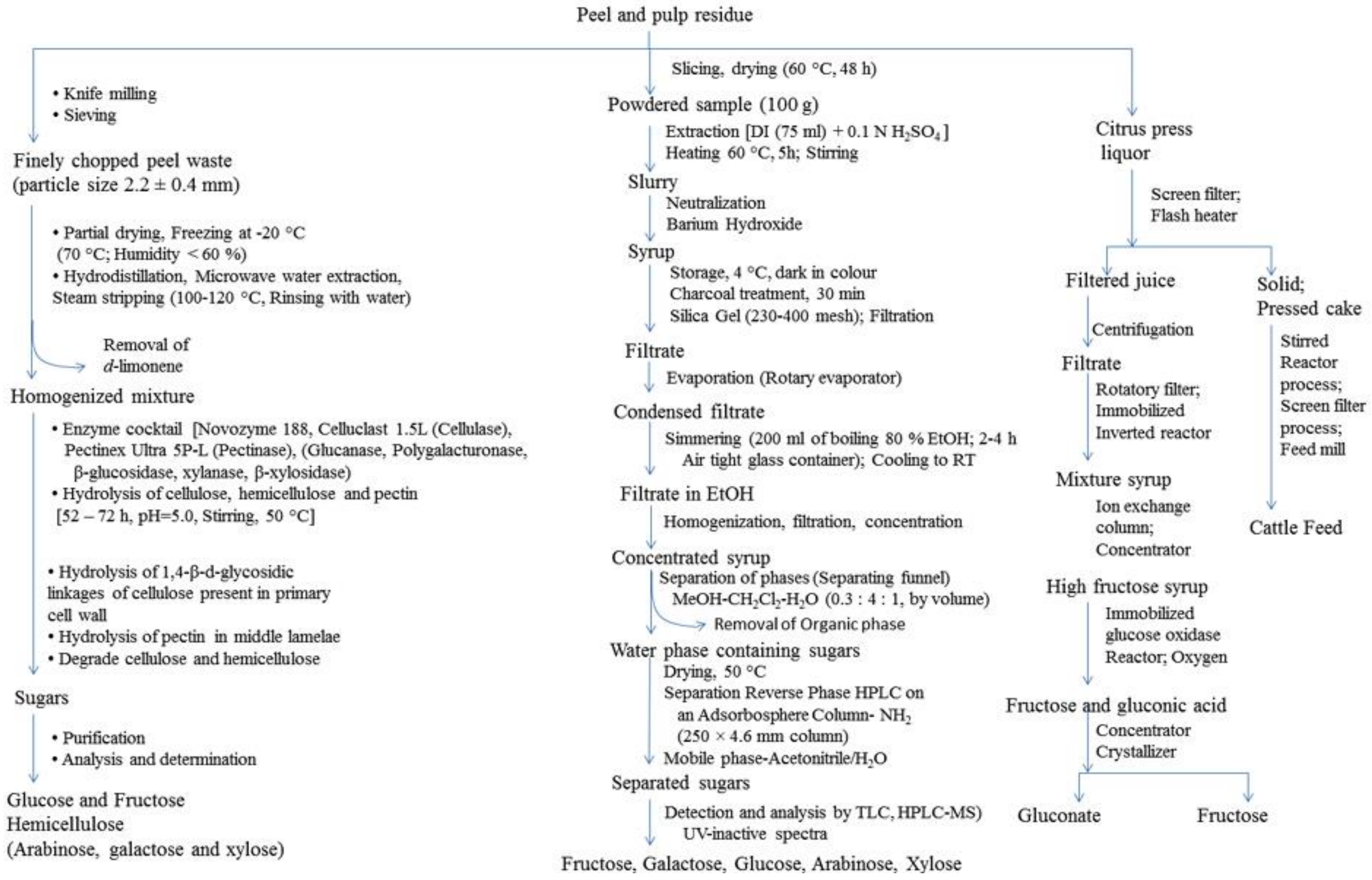

Figure S-22: Steps involved in the extraction of sugars from citrus waste [66, 86, 88, 89]

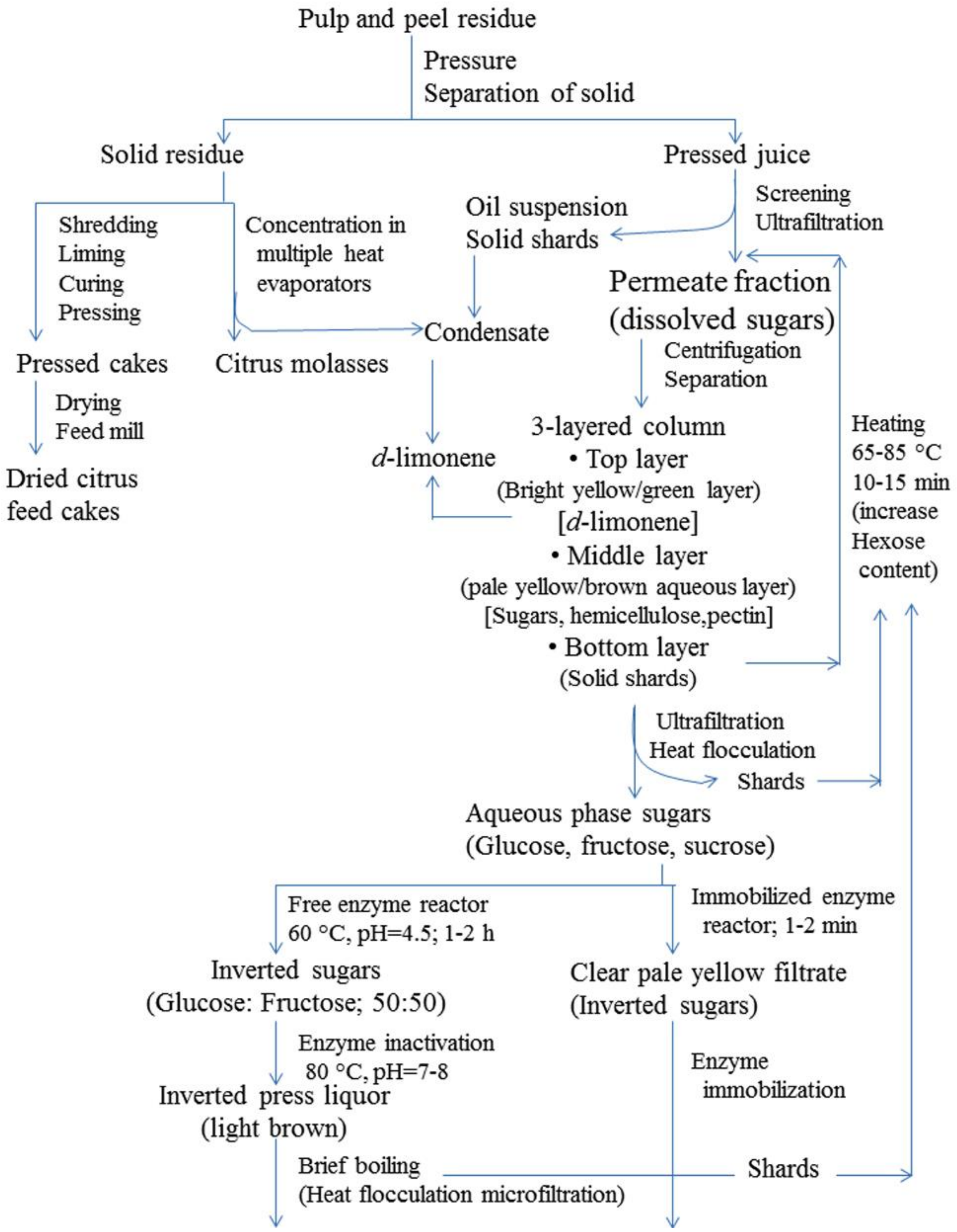
Pulp and peel residue
Pressure
Separation of solid
Solid residue
Pressed juice
Shredding
Liming
Curing
Pressing
Concentration in multiple heat evaporators
Oil suspension
Solid shards
Screening
Ultrafiltration
Permeate fraction (dissolved sugars)
Pressed cakes
Citrus molasses
Condensate
Centrifugation
Separation
Drying
Feed mill
Dried citrus feed cakes
d-limonene
3-layered column
• Top layer
(Bright yellow/green layer)
[d-limonene]
• Middle layer
(pale yellow/brown aqueous layer)
[Sugars, hemicellulose,pectin]
• Bottom layer
(Solid shards)
Heating
65-85 °C
10-15 min
(increase Hexose content)
Ultrafiltration
Heat flocculation
Shards
Aqueous phase sugars
(Glucose, fructose, sucrose)
Free enzyme reactor
60 °C, pH=4.5; 1-2 h
Immobilized enzyme reactor; 1-2 min
Inverted sugars
(Glucose: Fructose; 50:50)
Clear pale yellow filtrate
(Inverted sugars)
Enzyme inactivation
80 °C, pH=7-8
Enzyme immobilization
Inverted press liquor
(light brown)
Brief boiling
(Heat flocculation microfiltration)
Shards

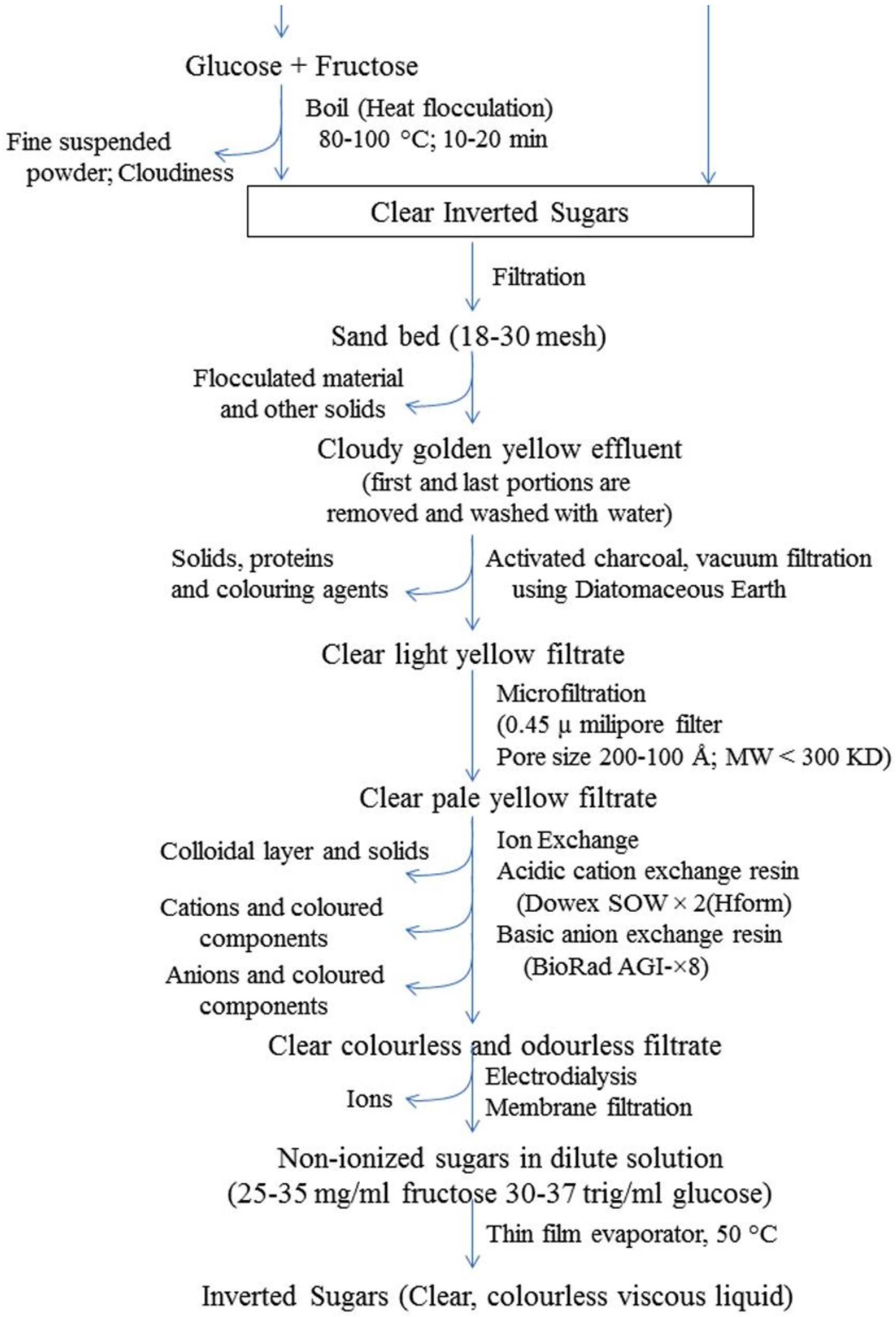

Figure S-23: Extraction of inverted sugars from citrus waste [90]

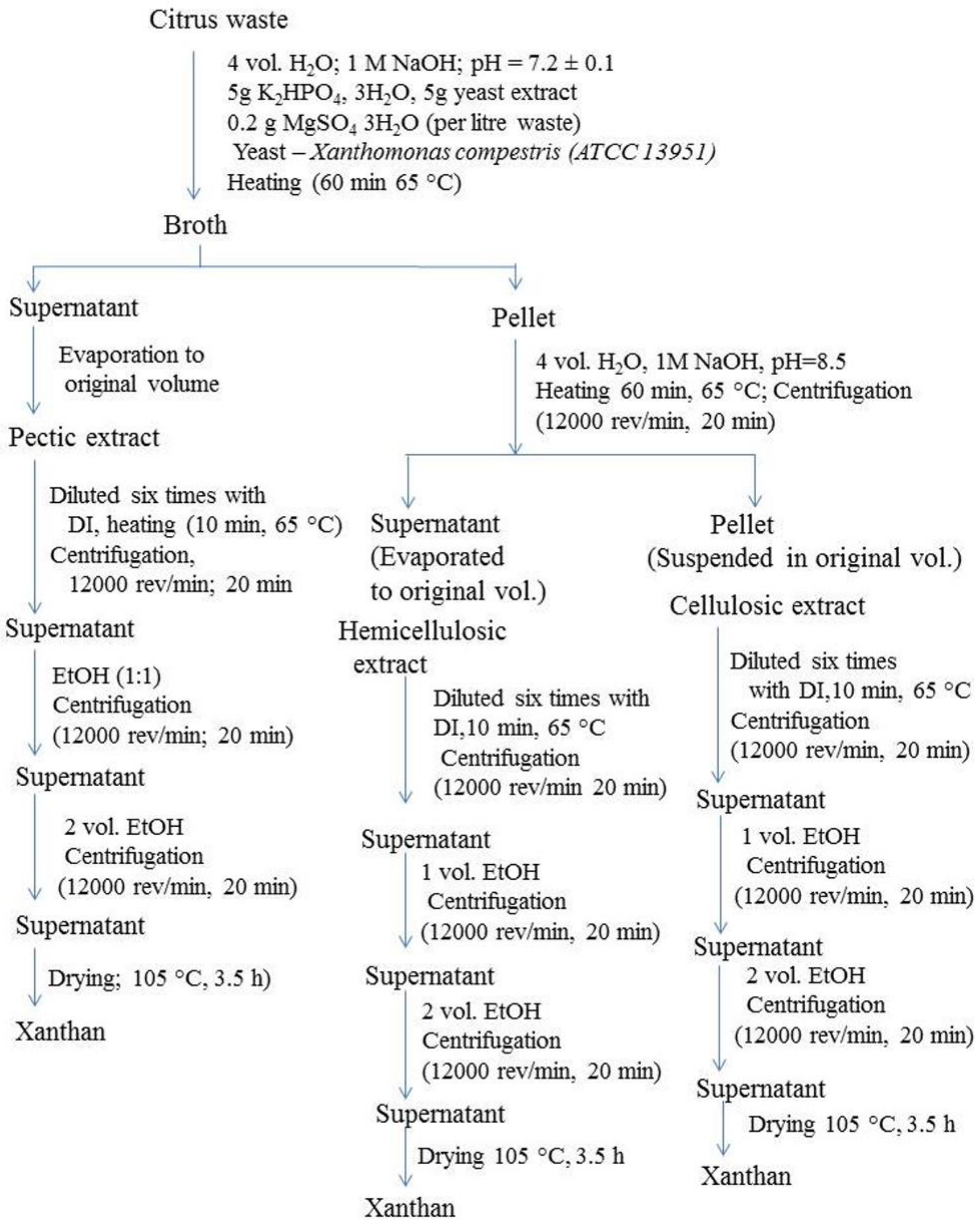

Figure S-24: Production of Xanthan gum from citrus waste [91]

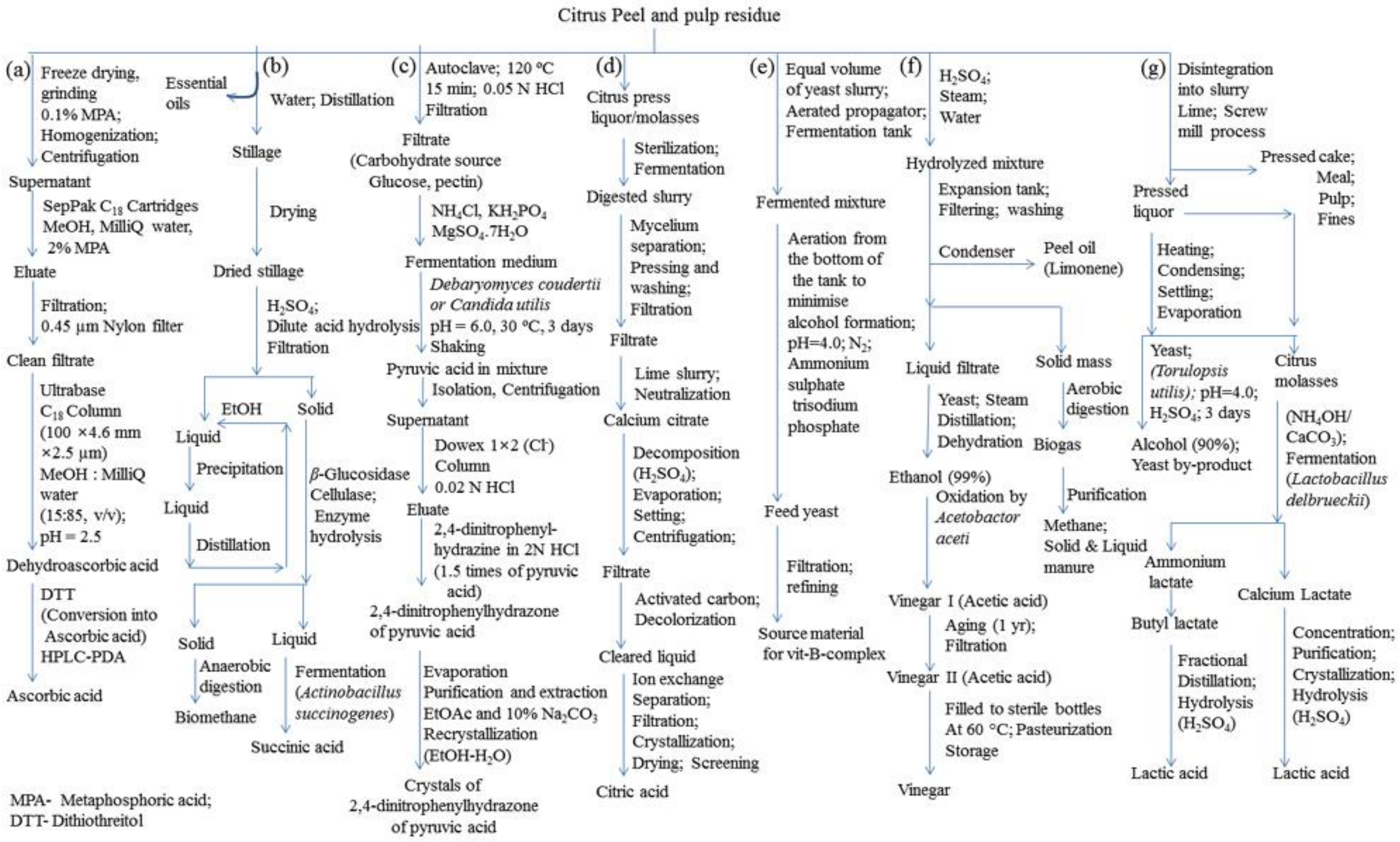

Figure S-25: Production of various important organic acids, *viz.*, succinic acid, citric acid, lactic acid and vinegar, and vitamins from citrus waste [66,86,92-96]

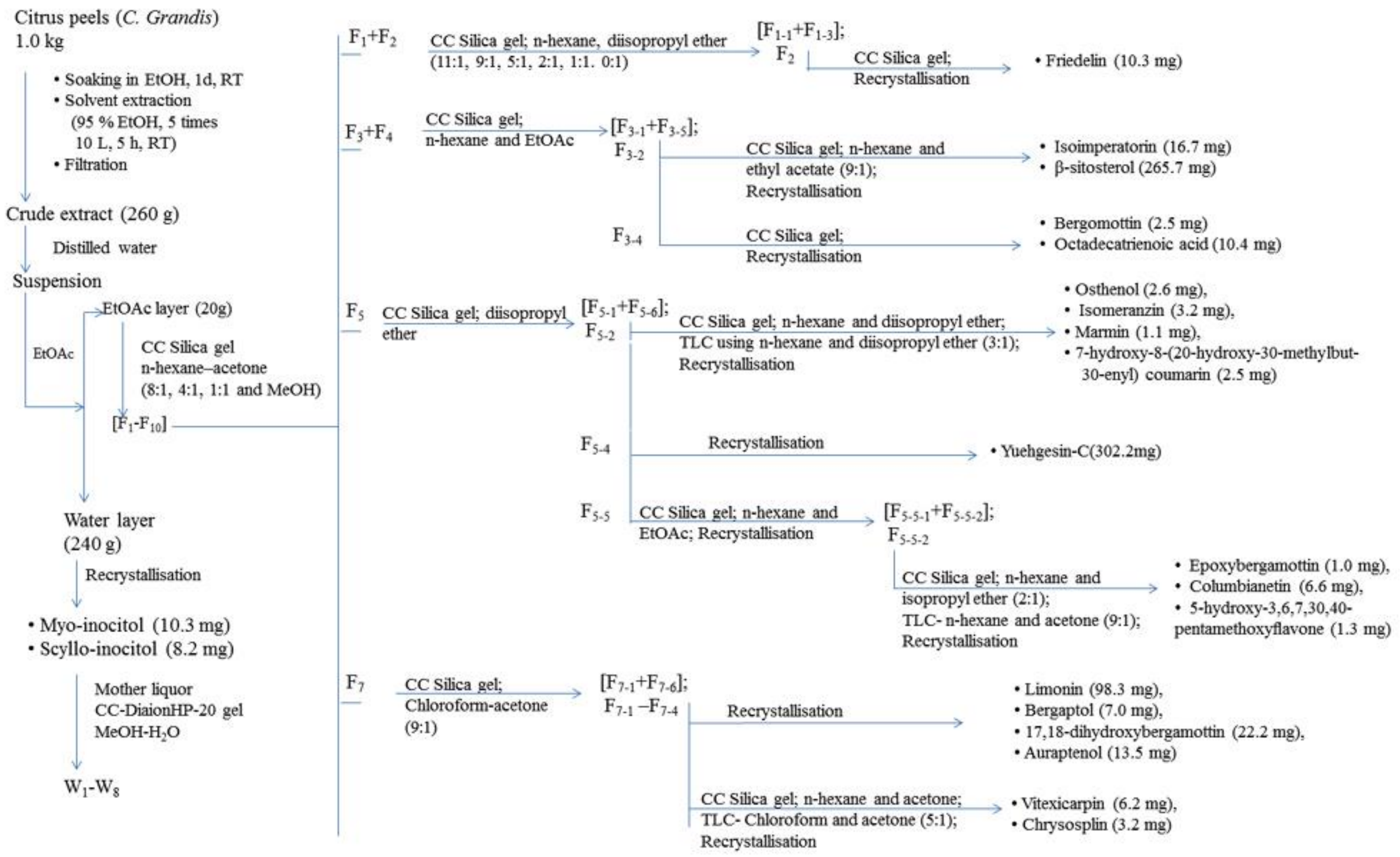

Citrus peels (*C. Grandis*) 1.0 kg
• Soaking in EtOH, 1d, RT
• Solvent extraction (95 % EtOH, 5 times 10 L, 5 h, RT)
• Filtration
Crude extract (260 g)
Distilled water
Suspension
EtOAc
EtOAc layer (20g)
CC Silica gel n-hexane–acetone (8:1, 4:1, 1:1 and MeOH)
[F_1-F_{10}]
Water layer (240 g)
Recrystallisation
• Myo-inocitol (10.3 mg)
• Scyllo-inocitol (8.2 mg)
Mother liquor CC-DiaionHP-20 gel MeOH-H_2O
W_1-W_8
F_1+F_2
CC Silica gel; n-hexane, diisopropyl ether (11:1, 9:1, 5:1, 2:1, 1:1. 0:1)
[F_{1-1}+F_{1-3}]; F_2
CC Silica gel; Recrystallisation
• Friedelin (10.3 mg)
F_3+F_4
CC Silica gel; n-hexane and EtOAc
[F_{3-1}+F_{3-5}]; F_{3-2}
CC Silica gel; n-hexane and ethyl acetate (9:1); Recrystallisation
• Isoimperatorin (16.7 mg)
• β-sitosterol (265.7 mg)
F_{3-4}
CC Silica gel; Recrystallisation
• Bergomottin (2.5 mg)
• Octadecatrienoic acid (10.4 mg)
F_5
CC Silica gel; diisopropyl ether
[F_{5-1}+F_{5-6}]; F_{5-2}
CC Silica gel; n-hexane and diisopropyl ether; TLC using n-hexane and diisopropyl ether (3:1); Recrystallisation
• Osthenol (2.6 mg),
• Isomeranzin (3.2 mg),
• Marmin (1.1 mg),
• 7-hydroxy-8-(20-hydroxy-30-methylbut-30-enyl) coumarin (2.5 mg)
F_{5-4}
Recrystallisation
• Yuehgesin-C(302.2mg)
F_{5-5}
CC Silica gel; n-hexane and EtOAc; Recrystallisation
[F_{5-5-1}+F_{5-5-2}]; F_{5-5-2}
CC Silica gel; n-hexane and isopropyl ether (2:1); TLC- n-hexane and acetone (9:1); Recrystallisation
• Epoxybergamottin (1.0 mg),
• Columbianetin (6.6 mg),
• 5-hydroxy-3,6,7,30,40-pentamethoxyflavone (1.3 mg)
F_7
CC Silica gel; Chloroform-acetone (9:1)
[F_{7-1}+F_{7-6}]; F_{7-1} –F_{7-4}
Recrystallisation
• Limonin (98.3 mg),
• Bergaptol (7.0 mg),
• 17,18-dihydroxybergamottin (22.2 mg),
• Auraptenol (13.5 mg)
CC Silica gel; n-hexane and acetone; TLC- Chloroform and acetone (5:1); Recrystallisation
• Vitexicarpin (6.2 mg),
• Chrysosplin (3.2 mg)

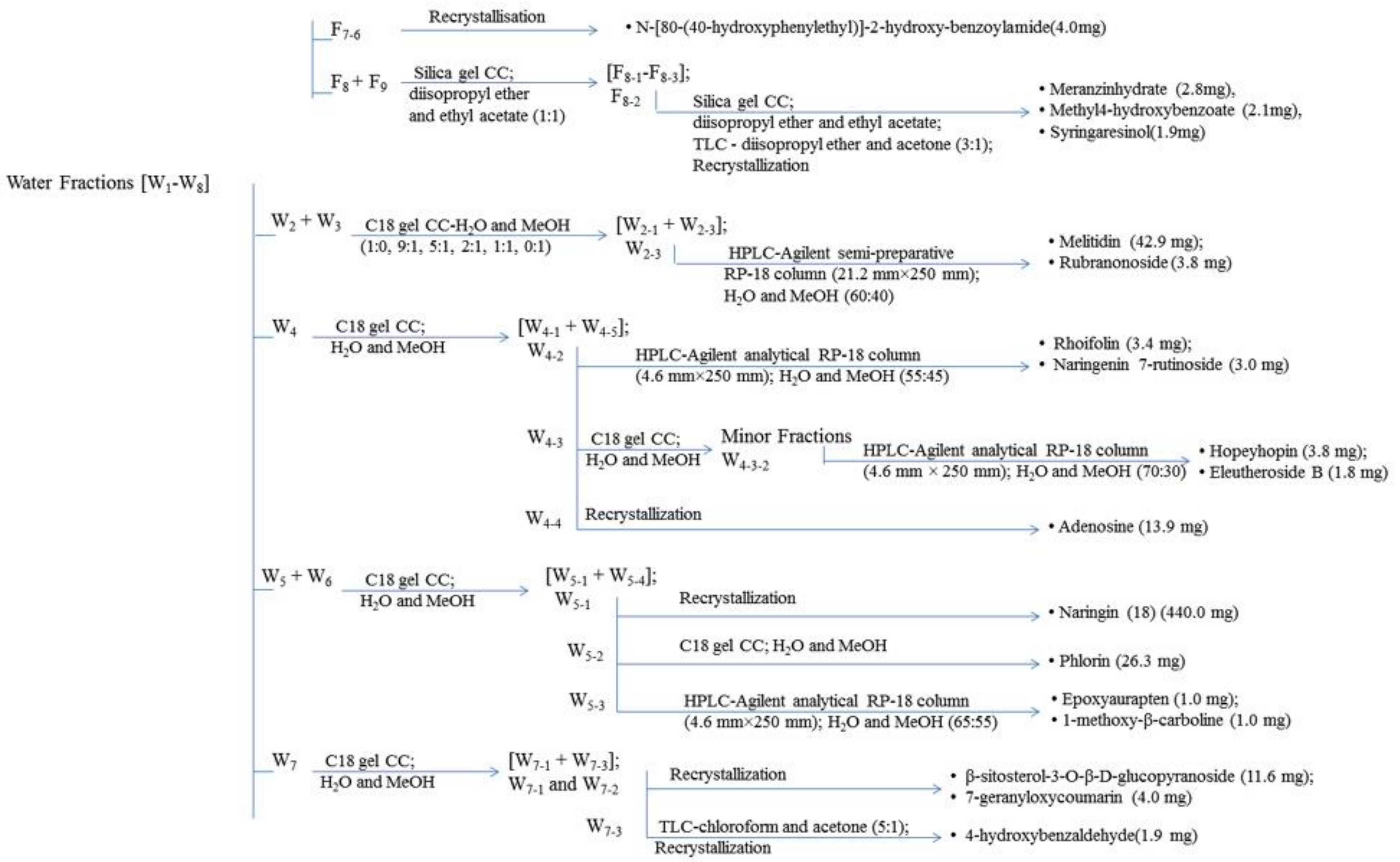

Figure S-26: Steps involved in the extraction, separation and isolation, and determination of different phenolic compounds [97]

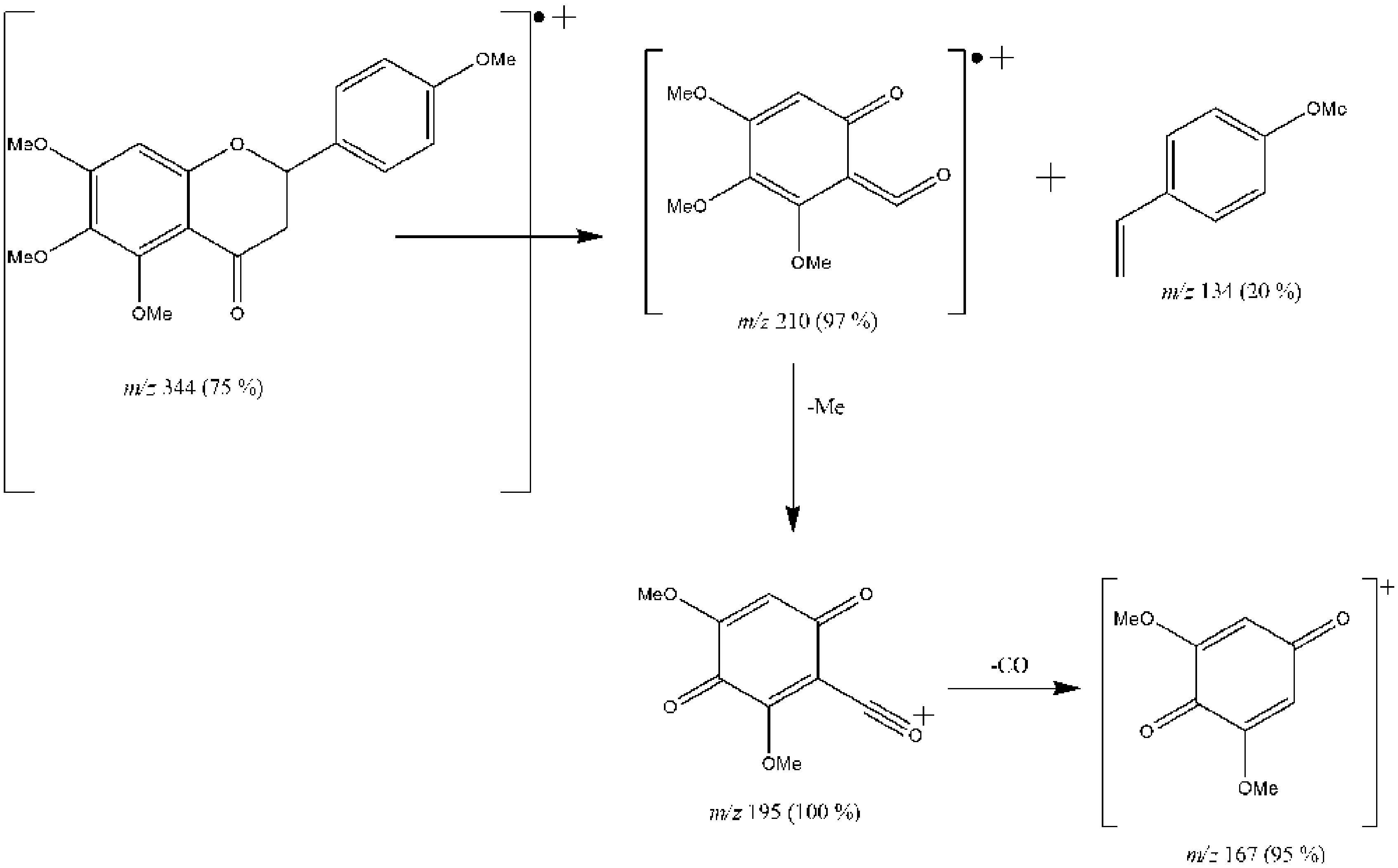

Figure S-27: Fragmentation pathway of 5,6,7,4′-tetramethoxyflavanone [98]

References:

1. Report. *Citrus fruits 2016 Summary*; United States Department of Agriculture, National Agricultural Statistics Service.

2. Agriculture, U.S.D.A. *Citrus: World markets and trade* Foreign Agricultural Service 2019 July.

3. Distribution, E. *The growth of the world's citrus trade*; Produce Citrus: 2019.

4. Wu, G.A.; Terol, J.; Ibanez, V.; López-García, A.; Pérez-Román, E.; Borredá, C.; Domingo, C.; Tadeo, F.R.; Carbonell-Caballero, J.; Alonso, R., et al. Genomics of the origin and evolution of Citrus. *Nature* **2018**, *554*, 311–316.

5. Weisskopf, A.; Fuller, D.Q. *Citrus fruits: Origins and development*; Springer: New York, NY, 2014.

6. Kefford, J.F. The chemical constituents of citrus fruits. *Advances in Food Research* **1960**, *9*, 285-372.

7. Azmir, J.; Zaidul, I.S.M.; Rahman, M.M.; Sharif, K.M.; Mohamed, A.; Sahena, F.; Jahurul, M.H.A.; Ghafoor, K.; Norulaini, N.A.N.; Omar, A.K.M. Techniques for extraction of bioactive compounds from plant materials: A review. *Journal of Food Engineering* **2013**, *117*, 426–436.

8. Liu, Y.; Shi, J.; Langrish, T.A.G. Water-based extraction of pectin from flavedo and albedo of orange peels. *Chemical Engineering Journal* **2006**, *120*, 203-209.

9. Vankar, P.S. Essential oils and fragrances from natural sources. *Resonance* **2004**, *9*, 30-41.

10. Silva, L.V.; Nelson, D.L.; Drummond, M.F.B.; Dufossé, L.; Glória, M.B.A. Comparison of hydrodistillation methods for the deodorization of turmeric. *Food Research International* **2005**, *38*, 1087-1096.

11. Suslick, K.S.; Doktycz, S.J. *The effects of ultrasound on solids" in Advances in Sonochemistry*; JAI Press: New York, 1990; Vol. 1, pp. 197-230.

12. Mason, T.J.; Paniwnyk, L.; Lorimer, J.P. The uses of ultrasound in food technology. *Ultrasonics Sonochemistry* **1996**, *3*, 253-260.

13. Nishad J.; Saha S.; Kaur C. Enzyme- and ultrasound-assisted extractions of polyphenols from *Citrus sinensis* (cv. Malta) peel: A comparative study. *Journal of Food Processing and Preservation* **2019**, 43, e14046.

14. Bryant, G.; Wolfe, J. Electromechanical stress produced in the plasma membranes of suspended cells by applied electrical fields. *Journal of Membrane Biology* **1987**, *96* 129-139.

15. Fincan, M.; Dejmek, P. *In situ* visualization of the effect of a pulsed electric field on plant tissue. *Journal of Food Engineering* **2002**, *55*, 223-230.

16. Lebovka, N.I.; Bazhal, M.I.; Vorobiev, E. Estimation of characteristic damage time of food materials in pulsed-electric fields. *Journal of Food Engineering* **2002**, *54*, 337-346.

17. Singh, R.K.; Sarker, B.C.; Kumbhar, B.K.; Agrawal, Y.C.; Kulshreshtha, M.K. Response surface analysis of enzyme-assisted oil extraction factors for sesame, groundnut, and sunflower seeds. *Journal of Food Science and Technology* **1999**, *36*, 511-514.

18. Latif, S.; Anwar, F. Physicochemical studies of hemp (*Cannabis sativa*) seed oil using enzyme-assisted cold-pressing. *European Journal of Lipid Science and Technology* **2009**, *111*, 1042-1048.

19. Hanmoungjai, P.; Pyle, D.L.; Niranjan, K. Enzymatic process for extracting oil and protein from rice bran. *Journal of the American Oil Chemists Society* **2001**, *78*, 817-821.

20. Rosenthal, A.; Pyle, D.L.; Niranjan, K.; Gilmour, S.; Trinca, L. Combined effect of operational variables and enzyme activity on aqueous enzymatic extraction of oil and protein from soybean. *Enzyme and Microbial Technology* **2001**, *28*, 499– 509.

21. Sharma, A.; Khare, S.K.; Gupta, M.N. Enzyme-assisted aqueous extraction of peanut oil. *Journal of American Oil Chemist's Society* **2002**, *79*, 215-218.

22. Concha, J.; Soto, C.; Chamy, R.; Zuniga, M.E. Enzymatic pretreatment on Rose- Hip oil extraction: hydrolysis and pressing conditions. *Journal of American Oil Chemist's Society* **2004**, *81*, 549-552.

23. Niranjan, K.; Hanmoungjai, P. *Enzyme-aided aqueous extraction*; ACS Publishing: 2004.

24. Dominguez, H.; Ntiiiez, M.J.; Lema, J.M. Enzyme-assisted hexane extraction of soybean oil. *Food Chemistry* **1995**, *54*, 223-231.

25. Puri, M.; Sharma, D.; Barrow, C.J. Enzyme-assisted extraction of bioactives from plants. *Trends in Biotechnology* **2012**, *30*, 37-44.

26. Jain, T. Microwave assisted extraction for phytoconstituents – An overview. *Asian Journal of Research in Chemistry* **2009**, *2*, 19-25.

27. Letellier, M.; Budzinski, H. Microwave assisted extraction of organic compounds. *Analysis* **1999**, *27*, 259-270.

28. Nieto, A.; Borrull, F.; Pocurull, E.; Marcé, R.M. Pressurized liquid extraction: a useful technique to extract pharmaceuticals and personal-care products from sewage sludge. *Trends in Analytical Chemistry* **2010**, *29*, 752-764.

29. Ibañez, E.; Herrero, M.; Mendiola, J.A.; Castro-Puyana, M. *Extraction and characterization of bioactive compounds with health benefits from marine resources: macro and micro algae, cyanobacteria, and invertebrates*; Springer: 2012; pp. 55-98.

30. Luengo, E.; Alvarez, I.; Raso, J. Improving the pressing extraction of polyphenols of orange peel by pulsed electric fields. *Innovative Food Science & Emerging Technologies* **2013**, *17*, 79-84.

31. Richter, B.E.; Jones, B.A.; Ezzell, J.L.; Porter, N.L.; Avdalovic, N.; Pohl, C. Accelerated solvent extraction: A technique for sample preparation. *Analytical Chemistry* **1996**, *68*, 1033-1039.

32. Hannay, J.B.; Hogarth, J. On the solubility of solid in gases. *Proceeding of the Royal Society of London* **1879**, *29*, 324-326.

33. Zosel, K. Method for separation of mixtures. 1964.

34. Sihvonen, M.; Järvenpää, E.; Hietaniemi, V.; Huopalahti, R. Advances in supercritical carbon dioxide technologies. *Trends in Food Science and Technology* **1999**, *10*, 217-222.

35. Giannuzzo, A.N.; Boggetti, H.c.J.; Nazareno, M.n.A.; Mishima, H.T. Supercritical fluid extraction of naringin from the peel of *Citrus paradisi*. *Phytochemical Analysis* **2003**, *14*, 221-223.

36. Herrero, M.; Mendiola, J.A.; Cifuentes, A.; Ibá˜nez, E. Supercritical fluid extraction: Recent advances and applications. *Journal of Chromatography A* **2010**, *1217*, 2495-2511.

37. Kim, J.-W.; Nagaoka, T.; Ishida, Y.; Hasegawa, T.; Kitagawa, K.; Lee, S.-C. Subcritical water extraction of nutraceutical, compounds from citrus pomaces. *Separation Science and Technology* **2009**, *44*, 2598-2608.

38. M'hiri, N.; Ioannou, I.; Ghoul, M.; Boudhrioua, N.M. Extraction methods of citrus peel phenolic compounds: a review. *Food Reviews International* **2014**, *30,* 265-290.

39. Ferhat, M.A.; Meklati, B.Y.; Farid Chemat. Comparison of different isolation methods of essential oil from Citrus fruits: cold pressing, hydrodistillation and microwave 'dry' distillation. *Flavour Fragrance Journal* **2007**, *22*, 494-504.

40. Shakir, I.K.; Salih, S.J. Extraction of essential oils from citrus by-products using microwave steam distillation. *Iraqi Journal of Chemical and Petroleum Engineering* **2015**, *16,* 11- 22.

41. Ferhat, M.A.; Boukhatem, M.N.; Hazzit, M.; Meklati, B.Y.; Chemat, F. Cold pressing, hydrodistillation and microwave dry distillation of citrus essential oil from Algeria: A comparative study. *Electronic Journal of Biology* **2016**, *S1*, 30-41.

42. Wilkins, M.R.; Widmer, W.W.; Grohmann, K. Simultaneous saccharification and fermentation of citrus peel waste by *Saccharomyces cerevisiae* to produce ethanol. *Process Biochemistry* **2007**, *42*, 1614-1619.

43. Yanxiang Gao; Nagy, B.; Liu, X.; Béla Simándi; Wang, Q. Supercritical CO_2 extraction of lutein esters from marigold (*Tagetes erecta* L.) enhanced by ultrasound. *Journal of Supercritical Fluids* **2009**, *49*, 345-350.

44. Bousbia, N.; Vian, M.A.; Ferhat, M.A.; Meklati, B.Y.; Chemat, F. A new process for extraction of essential oil from Citrus peels: Microwave hydrodiffusion and gravity. *Journal of Food Engineering* **2009**, *90*, 409-413.

45. Singleton, V.L.; Rosi, J.A. Colorimetry of total phenolics with phosphomolybdic– phosphotungstic acid reagents. *American Journal of Viticulture Enology* **1965**, *16*, 144–158.

46. Li, B.B.; Smith, B.; Hossain, M. Extraction of phenolics from citrus peels I.

Solvent extraction method. *Separation and Purification Technology* **2006**, *48*, 182–188.

47. Guihua Xu; Ye, X.; Chen, J.; Liu, D. Effect of heat treatment on the phenolic compounds and antioxidant capacity of citrus peel extract. *Journal of Agricultural and Food Chemistry* **2007**, *55*, 330-335.

48. Prieto, P.; Pineda, M.; Aguilar, M. Spectrophotometric quantitation of antioxidant capacity through the formation of a phosphomolybdenum complex: specific application to the determination of vitamin E. *Analytical Biochemistry***1999**, *269*, 337-341.

49. Tumbas, V.T.; Ćetković, G.S.; Djilas, S.M.; Čanadanović-Brunet, J.M.; Vulić, J.J.; Knez, Ž.; Škerget, M. Antioxidant activity of mandarin (*Citrus reticulata*) peel. *Acta periodica technologica* **2010**, *41*, 1-203.

50. Dhuique-Mayer, C.; Tbatou, M.; Carail, M.; Caris-Veyrat, C.; Dornier, M.; Amiot, M.J. Thermal degradation of antioxidant micronutrients in citrus juice: kinetics and newly formed compounds. *Journal of Agricultural and Food Chemistry* **2007**, *55*, 4209-4216.

51. Yerlikayaa, P.; Gokoglu, N.; Topuz, O.K.; Gumus, B.; Yatmaz, H.A. Antioxidant activities of citrus albedo and flavedo fragments against fish lipid oxidation. *Journal of Aquatic Food Product Technology* **2016.**

52. Safdar, M.N.; Kausar, T.; Jabbar, S.; Mumtazb, A.; Ahad, K.; Saddozai, A.A. Extraction and quantification of polyphenols from kinnow (*Citrus reticulata* L.) peel using ultrasound and maceration techniques. *Journal of Food and Drug Analysis* **2016**, 1-13.

53. Choi, S. Lipolytic effects of citrus peel oils and their components. *Journal of Agricultural and Food Chemistry* **2006**, *54*, 3254-3258.

54. Oong, S.H. Environmental friendly pollution -proof agent made from citrus fruits. 2006.

55. Hussain.A.Abd El-aal, F.T.H. Food preservative activity of phenolic compounds in orange peel extracts (*Citrus sinensis* L.). *LucrăriŞtiinţifice Seria Zootehnie* **2010**, *53*, 233-240.

56. Bhatlu, M.L.D.; Prashant Katiyar; Singh, S.; Verma, A.K. Pre-harvest dropped kinnow (*Citrus reticulata* Blanco) waste management through the extraction of

naringin and pectin from their peels using indigenous resin. *Journal of the Institution of Engineers (India): Series A* **2016**, *97*, 285-290.

57. MaríaBoluda-Aguilar; García-Vidal, L.; González-Castañeda, F.d.P.; López-Gómez, A. Mandarin peel wastes pretreatment with steam explosion for bioethanol production. *Bioresource Technology* **2010**, *101*, 3506–3513.

58. Zouambia, Y.; Ettoumi, K.Y.; Krea, M.; Moulai-Mostefa, N. A new approach for pectin extraction: Electromagnetic induction heating. *Arabian Journal of Chemistry* **2017**, *10*, 480–487.

59. Belisario-Sánchez, Y.Y.; A.Taboada-Rodríguez; Marín-Iniesta, F.; López-Gómez, A. Dealcoholized wines by spinning cone column distillation: phenolic compounds and antioxidant activity measured by the 1,1-diphenyl-2-picrylhydrazyl method. *Journal of Agricultural and Food Chemistry* **2009**, *57*, 6770–6778.

60. MaríaBoluda-Aguilar; López-Gómez, A. Production of bioethanol by fermentation of lemon (*Citrus limon* L.) peel wastes pretreated with steam explosion. *Industrial Crops and Products* **2013**, *41*, 188– 197.

61. Quoc, L.P.T.; Huyen, V.T.N.; Hue, L.T.N.; Hue, N.T.H.; Thuan, N.H.D.; Tam, N.T.T.; Thuan, N.N.; Duy, T.H. Extraction of pectin from pomelo (*Citrus maxima*) peels with the assistance of microwave and tartaric acid. *International Food Research Journal* **2015**, *22*, 1637-1641.

62. Goodrich, R.M.; Braddock, R.J. *Major by-products of the Florida Citrus Processing Industry*; Food Science and Human Nutrition Department, Florida Cooperative Extension Service, Institute of Food and Agricultural Sciences, University of Florida: Florida, FSHN05-22, 2006.

63. Hegde, P.; Agrawal, P.; Gupta, P.K. Isolation and optimization of polyphenols from the peels of orange fruit. *Journal of Chemical and Pharmaceutical Sciences* **2015**, *8*, 463-468.

64. Khan, M.K.; Abert-Vian, M.; Fabiano-Tixier, A.S.; Dangles, O.; Chemat, F. Ultrasound-assisted extraction of polyphenols (flavanone glycosides) from orange (*Citrus sinensis* L.) peel. *Food Chemistry* **2010**, *119*, 851-858.

65. Xu, G.; Ye, X.; Chen, J.; Liu, D. Effect of heat treatment on the phenolic compounds and antioxidant capacity of citrus peel extract. *Journal of Agricultural and Food chemistry* **2007**, *55*, 330-335.

66. Shan, Y. *Comprehensive utilization of citrus by-products*; Academic Press Elsevier: London, UK, 2016.

67. Nafisi-Movaghar, K.; Druz, L.L.; Victoria, C.P. Process for conversion of citrus peels into fiber, juice, naringin and oil. US 8481099B2 (Patent) 2013.

68. Service, A.R. *Chemistry and technology of citrus, citrus products and byproducts*; US Government Printing Office: Washington DC, 1956.

69. Rosa J. D.P; Ruiz-Palomino P.; Arriola-Guevara E.; García-Fajardo J.; Sandoval G.; Guatemala-Morales G.M. A green process for the extraction and purification of hesperidin from Mexican lime peel (*Citrus aurantifolia* Swingle) that is extendible to the Citrus genus. *Processes* **2018**, 6, 266(1-13).

70. Hoshino, M.; Suetsugu, T.; Iwai, H.; Takamizu, A.; Tanaka, M.; Quitain, A.; Sasaki, M.; Goto, M. Extraction of citrus flavonoids from peel of *Citrus junos* using supercritical carbon dioxide with polar solvent. *Transactions of the Materials Research Society of Japan* **2014**, *39*, 309-311.

71. Nandan, M.P.; Meena, V. Extraction, modelling and purification of flavonoids from *Citrus medica* peel. *International Journal of Applied Sciences and Biotechnology* **2015**, *3*, 588-591.

72. Londoño-Londoño, J.; Lima, V.R.d.; Lara, O.; Gil, A.; Pasa, T.B.C.; Arango, G.J.; Pineda, J.R.R. Clean recovery of antioxidant flavonoids from citrus peel: Optimizing an aqueous ultrasound-assisted extraction method. *Food Chemistry* **2010**, *119*, 81-87.

73. Inoue, T.; Tsubaki, S.; Ogawa, K.; Onishi, K.; Azuma, J.-i. Isolation of hesperidin from peels of thinned *Citrus unshiu* fruits by microwave-assisted extraction. *Food Chemistry* **2010**, *123*, 542-547.

74. Cheigh, C.I.; Chung, E.Y.; Chung, M.S. Enhanced extraction of flavanones hesperidin and narirutin from *Citrus unshiu* peel using subcritical water. *Journal of Food Engineering* **2012**, *110*, 472-477.

75. Magwaza, L.S.; Oara, U.L.; Cronje, P.j.R.; Landahl, S.; Ortiz, J.O.; Terry, L.A. Rapid methods for extracting and quantifying phenolic compounds in citrus rinds. *Food Science and Nutrition* **2016**, *4*, 4-10.

76. Li, B.B.; Smith, B.; Hossain, M.M. Extraction of phenolics from citrus peels. I. Solvent extraction method. *Separation and Purification Technology* **2006**, *48*, 182–

188.

77. Bocco, A.; Cuvelier, M.-E.; Richard, H.; Berset, C. Antioxidant activity and phenolic composition of citrus peel and seed extracts. *Journal of Agricultural and Food Chemistry* **1998**, *46*, 2123-2129.

78. Sharma, K.; Mahato, N.; Cho, M.H.; Lee, Y.R. Converting citrus wastes into value added products: Economical and environment friendly approaches. *Nutrition* **2017**, *34*, 29-46.

79. Patil, B.S.; Yu, J.; Dandekar, D.V.; Toledo, R.T.; Singh, R.K.; Pike, L.M. Citrus bioactive limonoids and flavonoids extraction by supercritical fluids. *Potential Health Benefits of Citrus, ACS Symposium Series* **2006**, *936*, 18-33.

80. Jayaprakasha, G.K.; Brodbelt, J.S.; BhatBhimanagouda, N.G.; Patil, S. Methods for the separation of limonoids from citrus. In Proceedings of Potential Health Benefits of Citrus; pp. 34-51.

81. Andrade, A.S.; Schmitt, G.C.; Rossato, L.G.; Russowsky, D.; Limberger, P. Gas chromatographic method for analysis of *p*-synephrine in *Citrus aurantium* L. products. *Chromatographia* **2009**, *69*, 225-229.

82. Johnson, J.D.; Viale, H.E.; Wait, D.M. Method for extracting carotenoid pigments from citrus oils. 1978.

83. Shukla, P.; Gawande, S.; Thorat, A. Extraction, Identification and Utilization of Pigments extracted from citrus wastes. *International Journal of Science and Research* **2016**, *5*, 840-847.

84. Boukroufa, M.; Boutekedjiret, C.; Chemat, F. Development of a green procedure of citrus fruits waste processing to recover carotenoids. *Resource-Efficient Technologies* **2017**, *3*, 252–262.

85. Johnson, J.D.; Viale, H.E.; Wait, D.M. Method for extracting carotenoid pigments from citrus oils. 1977.

86. Service), A.A.R. *Chemistry and technology of citrus, citrus products and byproducts*; United States Department of Agriculture: Washington, DC, 1962.

87. Hiasa, S.; Iwamoto, S.; Endo, T.; Edashige, Y. Isolation of cellulose nanofibrils from mandarin (*Citrus unshiu*) peel waste. *Industrial Crops and Products* **2014**, *62*, 280–285.

88. Kumar, C.S.C.; Mythily, R.; Chandraju, S. Extraction of carbohydrate from sweet orange peels (*Citrus sinensis* L.) and their identification via LC /MS and thin layer chromatographic analysis. *Biosciences Biotechnology Research Asia* **2011**, *8*, 709-715.

89. Velasco, D.; Senit, J.J.; Torre, I.d.l.; Santos, T.M.W.; Yustos, P.; Santos, V.E.; Ladero, M. Optimization of the enzymatic saccharification process of milled orange wastes. *Fermentation* **2017**, *3*, 37.

90. Milch, R.A.; Guerry-Kopecko, P.; Koeble-Smith, C.; Sybert, E.M. Preparation of high fructose syrups from citrus residues. 1985.

91. Bilanovic, D.; Shelef, G.; Green, M. Xanthan fermentation of citrus waste. *Bioresource Technology* **1994**, *48*, 169-172.

92. Moriguchi, M. Fermentative production of pyruvic acid from citrus peel extract by *Debaryomyces coudertii*. *Agricultural and Biological Chemistry* **1982**, *46*, 955 -961.

93. Oguntoyinbo, S.I.; Babajide, J.M.; Adenekan, M.K.; Ajayi, J.O.; Kareem, S.O.; Ayelaagbe, I.O.; Atanda, O.O.; Bodunde, G. Chemical properties of vinegar produced from sweet orange peels (*Citrus sinensis*). *Journal of Agricultural and Veterinary Sciences* **2011**, *3*, 51-61.

94. Torrado, A.M.; Cortés, S.; Salgado, J.M.; Max, B.; Rodríguez, N.; Bibbins, B.P.; ConvertiI, A.; Domínguez, J.M. Citric acid production from orange peel wastes by solid-state fermentation. *Brazilian Journal of Microbiology* **2011**, *42*, 394-409.

95. Kagan, J.J.; Pilnik, W.; Smith, M.D. Citrus by-products, lactic acid production by fermentation of citrus peel juice. *Journal of Agricultural and Food Chemistry* **1960**, *8*, 236-238.

96. Patsalou, M.; Menikea, K.K.; Makri, E.; Vasquez, M.I.; Drouza, C.; Koutinas, M. Development of a citrus peel-based biorefinery strategy for the production of succinic acid. *Journal of Cleaner Production* **2017**, *166*, 706-716.

97. Kuo, P.-C.; Liao, Y.-R.; Hung, H.-Y.; Chuang, C.-W.; Hwang, T.-L.; Huang, S.-C.; Shiao, Y.-J.; Kuo, D.-H.; Wu, T.-S. Anti-inflammatory and neuroprotective constituents from the peels of *Citrus grandis*. *Molecules* **2017**, *22*, 967-967.

98. Li, S.; Lo, C.Y.; Ho, C.T. Hydroxylated polymethoxyflavones and methylated

flavonoids in sweet orange (*Citrus sinensis*) peel. *Journal of Agricultural and Food Chemistry* **2006**, *54*, 4176-4185.
